# Choroid plexus remodeling associated with inflammatory CSF exposure in preterm acquired hydrocephalus

**DOI:** 10.1016/j.isci.2026.117759

**Published:** 2026-10-01

**Authors:** Gio Jison, Noah Ramos, Fernando Cotrim-Gomes, Seunghyun Lee, Brian W. Hanak, Michael Muhonen, Leandro Castañeyra-Ruiz

**Affiliations:** 1 CHOC Children’s Research Institute, CHOC Children’s Hospital, Orange, CA 92868, USA; 2 Division of Neurosurgery, CHOC Children’s Hospital, Orange, CA 92868, USA; 3 Department of Pharmacology, University of La Laguna, San Cristóbal de La Laguna, Tenerife, Spain; 4 Department of Neurosurgery, University of California, Irvine, Irvine, CA, USA

**Keywords:** posthemorrhagic hydrocephalus, preterm infants, choroid plexus, choroid plexus remodeling, inflammatory cerebrospinal fluid, growth factor signaling, EGFR, VEGFR, MRI morphometry, neuroinflammation

## Abstract

Acquired hydrocephalus in preterm infants follows intraventricular hemorrhage (IVH) or central nervous system infection and is associated with inflammatory cerebrospinal fluid (CSF) profiles. We integrated clinical magnetic resonance imaging (MRI), CSF cytokine profiling, primary human choroid plexus (ChP) epithelial and endothelial cell assays, and independent transcriptomic and proteomic datasets to investigate ChP remodeling. Infants requiring CSF diversion exhibited greater ChP thickness independent of gestational age, ventricular size, and IVH severity, supporting its potential as a biomarker. Growth factor-enriched inflammatory CSF increased proliferation in primary human ChP epithelial and endothelial cells; EGFR and VEGFR inhibition attenuated these responses, and recombinant epidermal growth factor (EGF) and vascular endothelial growth factor (VEGF) reproduced proliferative effects. Independent IVH transcriptomic data confirmed Mki67 elevation in IVH ChP tissue. Integrated proteomic trajectories support a temporally dynamic remodeling framework linking acute inflammatory signaling to proliferative ChP activation and structural remodeling.

## Highlights

• Choroid plexus thickness increases in infants requiring CSF diversion
• Inflammatory CSF increases proliferation in human ChP epithelial cells
• Inflammatory CSF also promotes proliferation in human ChP endothelial cells
• EGFR and VEGFR inhibition attenuates inflammatory ChP proliferation

## Graphical abstract

**Figure:**
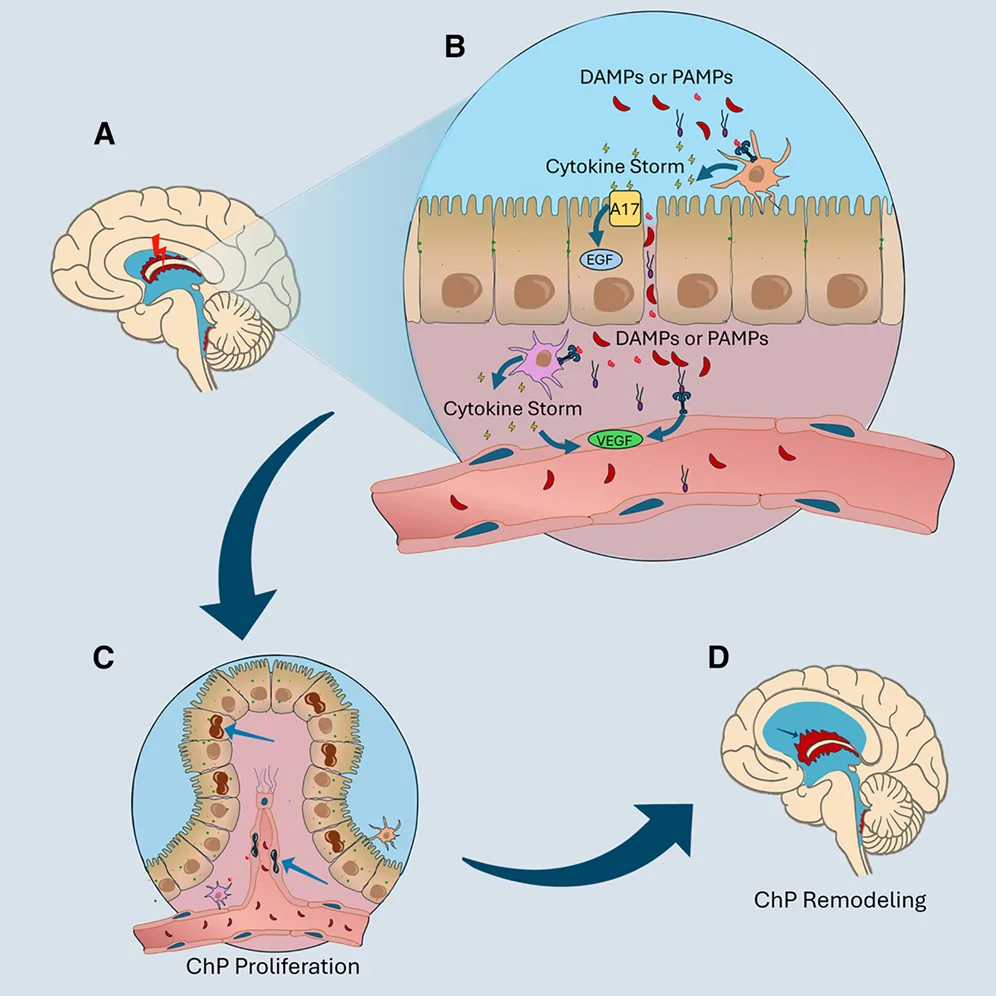

## Teaser

Neurology; Disease; Medical imaging

## Introduction

Pediatric acquired hydrocephalus remains a major cause of long-term neurological morbidity in preterm infants and most commonly develops following intraventricular hemorrhage (IVH) or central nervous system infection during the neonatal period^1^^,^^2^^,^^3^ Although these entities are traditionally categorized as post-hemorrhagic hydrocephalus (PHH) and post-infectious hydrocephalus (PIH), both occur disproportionately in very preterm infants,^4^^,^^5^^,^^6^^,^^7^ suggesting that developmental state plays an important role in disease susceptibility. Despite extensive characterization of inflammatory cerebrospinal fluid (CSF) profiles in these conditions, the cellular mechanisms linking early inflammatory exposure to structural changes within the choroid plexus (ChP) remain incompletely defined.

In PHH, inflammatory CSF signatures have been consistently documented. Preterm infants who progress to hydrocephalus following IVH exhibit inflammatory CSF signatures characterized by elevated cytokines and growth factors, including VEGF, IL-6, TNF-α, and related mediators.^6^^,^^8^ Growth factor enrichment has been variably reported across cohorts. Systematic analyses further underscore enrichment of cytokines and growth factors across diverse hydrocephalus etiologies,^9^ and neonatal profiling studies demonstrate etiology-specific inflammatory fingerprints.^10^ These inflammatory profiles correlate with clinical severity and have been associated with increased ChP size on magnetic resonance imaging (MRI).^11^^,^^12^ In PIH, neonatal cytokine datasets are more limited because many infants present after resolution of the acute infectious phase^6^; however, pediatric and experimental studies demonstrate that bacterial and viral CNS infections elicit VEGF- and EGF-linked inflammatory cascades, and survivors frequently develop ventricular enlargement.^13^ Together, these findings suggest that distinct initiating insults may converge on shared inflammatory growth-factor pathways in neonatal acquired hydrocephalus. Recent mechanistic reviews further support the concept that post-IVH hydrocephalus involves convergent inflammatory, glymphatic, and CSF regulatory disturbances rather than a purely obstructive process^7^

Innate immune activation likely represents an upstream event connecting hemorrhage or infection to downstream cytokine and growth-factor signaling. Blood-derived damage-associated molecular patterns (DAMPs) in IVH and pathogen-associated molecular patterns (PAMPs) during neonatal infection activate epiplexus and stromal macrophages within the ChP,^13^ generating a VEGF- and EGF-enriched inflammatory milieu.^14^ However, inflammatory signaling occurs within a developmental context. Increasing evidence suggests that inflammatory injury during periods of active developmental plasticity can intersect with intrinsic growth programs, potentially influencing tissue structure.^15^^,^^16^ Prematurity provides a particularly relevant biological setting for this interaction. Recent work has characterized meningeal lymphatic drainage as a critical CSF clearance route in central nervous system disease,^17^ described CSF transport mechanisms at the brain-meningeal border and their implications for neurological health,^18^ and demonstrated that therapeutic enhancement of glymphatic-meningeal lymphatic drainage alleviates hydrocephalus following IVH.^19^

During late gestation—approximately 24–28 weeks of human development—the epithelial and endothelial compartments of the ChP remain structurally immature and retain proliferative competence.^20^ At this stage, ChP tissue expresses growth-factor receptors including IGF-1R, EGFR, and VEGFR and responds to IGF-1, EGF, and VEGF signaling.^20^^,^^21^ Experimental exposure to these growth factors can stimulate ChP cell proliferation and tissue expansion *in vitro* and in explant cultures.^21^^,^^22^ Thus, inflammatory growth-factor enrichment during prematurity may intersect with developmentally active proliferative pathways.

Consistent with this concept, MRI studies report increased ChP thickness—a surrogate of ChP size—in infants with severe IVH and in neonatal CNS infections accompanied by strong inflammatory CSF profiles.^23^^,^^24^^,^^25^ Although MRI cannot distinguish epithelial proliferation from vascular remodeling, edema, or composite structural changes, the reproducible association between inflammatory states and ChP enlargement across neonatal and adult inflammatory conditions suggests that inflammatory signaling may influence ChP structure during periods of developmental susceptibility ADDIN EN.CITE.^25^^,^^26^

Growth-factor signaling provides a plausible mechanistic bridge between inflammatory exposure and ChP structural changes. VEGF and EGF are consistently elevated in inflammatory CSF associated with neonatal hydrocephalus^27^^,^^28^ and are established regulators of proliferation, angiogenesis, and epithelial remodeling in immature tissues.^29^^,^^30^ During prematurity, growth-factor receptor expression and downstream proliferative pathways remain relatively active,^31^^,^^32^^,^^33^ raising the possibility that inflammatory CSF may engage these programs in immature ChP cells.

Although severe IVH is a major risk factor for acquired hydrocephalus in preterm infants, only a subset of affected neonates ultimately progress to persistent hydrocephalus requiring permanent CSF diversion. Clinical management frequently involves temporary CSF drainage strategies, yet reliable biomarkers capable of predicting progression to shunt dependency remain limited.^34^^,^^35^ This unmet need highlights the importance of identifying biologically informative imaging markers associated with disease progression and inflammatory remodeling.

While inflammatory transporter activation and impaired resorption have been emphasized in models of hydrocephalus progression, the cellular basis of ChP enlargement observed on MRI remains uncertain. We therefore hypothesized that inflammatory CSF exposure during prematurity is associated with activation of growth factor–responsive proliferative programs within the immature ChP, providing a plausible cellular explanation for ChP enlargement observed in infants who progress to hydrocephalus.

To test this hypothesis, we integrated clinical neuroimaging, CSF inflammatory profiling, and *in vitro* experiments using primary human ChP epithelial and endothelial cells. By examining ChP size in infants with IVH and evaluating proliferative responses to CSF categorized by inflammatory burden, we sought to determine whether inflammatory CSF exposure is associated with growth factor-dependent proliferative activation in human ChP cells and structural enlargement detectable on MRI.

## Results

### Characterization of CSF used for experimental exposure

Multiplex cytokine analysis was performed to verify the growth factor profile of cerebrospinal fluid (CSF) samples selected for *in vitro* exposure. Because variance heterogeneity was detected (Brown-Forsythe *p* < 0.05), group comparisons were conducted using Welch’s ANOVA with Games-Howell post hoc testing.

VEGF concentrations differed significantly across groups (*p* < 0.001), with both IVH- and TBI-derived CSF demonstrating higher levels relative to SMA controls and no significant difference between IVH and traumatic brain injury (TBI) samples. Although EGF showed an overall group effect (*p* < 0.01), adjusted pairwise comparisons were not significant; given limited TBI sample size (*n* = 2) and variability, EGF was not used to determine exposure selection (Figure 1A).

**Figure 1:**
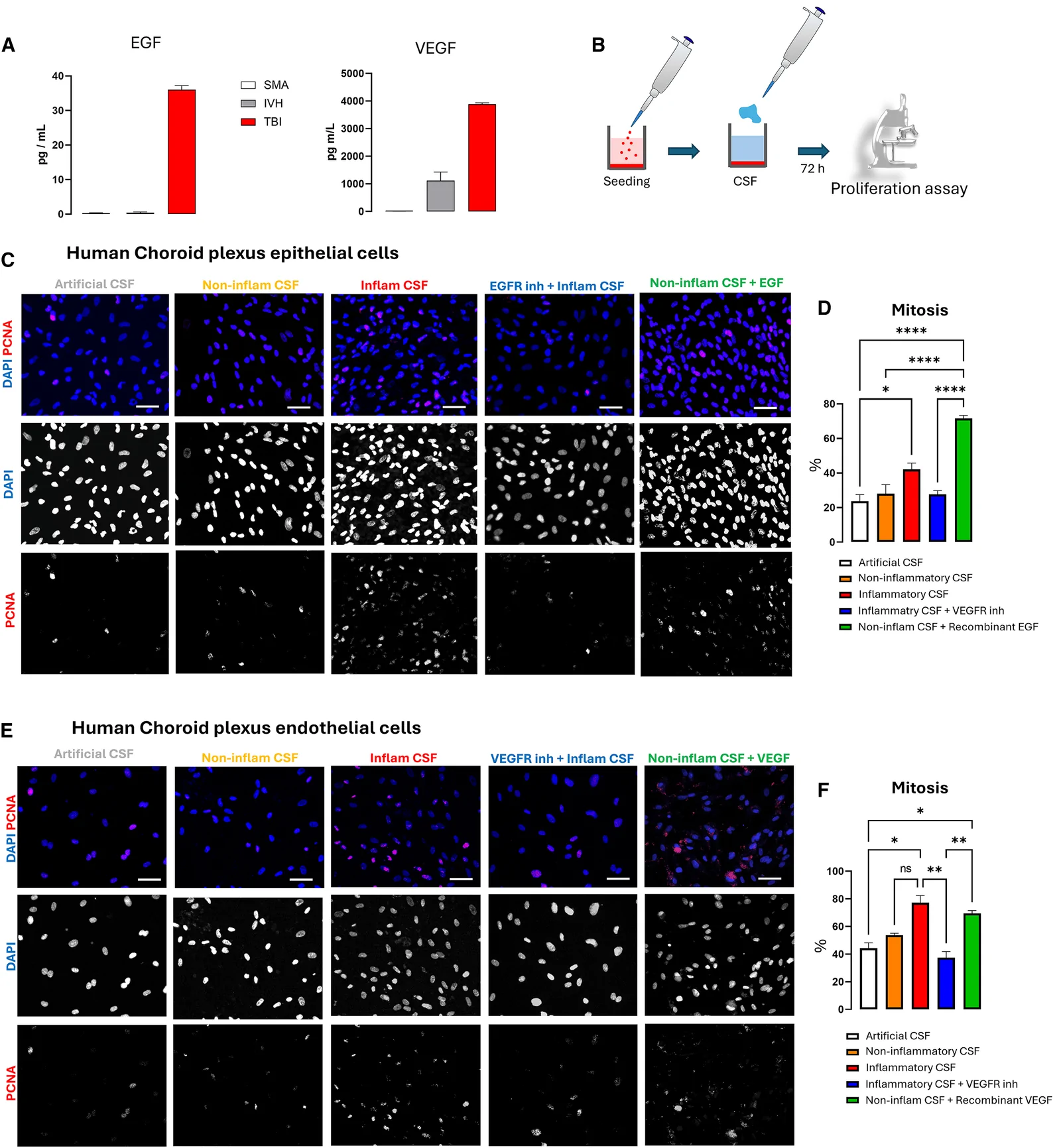
Inflammatory CSF induces growth factor-dependent proliferation in human choroid plexus epithelial and endothelial cells (A) Concentrations of epidermal growth factor (EGF) and vascular endothelial growth factor (VEGF) measured in cerebrospinal fluid (CSF) samples from non-inflammatory control (SMA), intraventricular hemorrhage (IVH), and traumatic brain injury (TBI) cohorts using multiplex Luminex analysis. Data are presented as mean + SD; SMA (*n* = 7), IVH (*n* = 4), TBI (*n* = 2). Statistical comparisons were performed using Welch’s ANOVA with Games-Howell post hoc testing. (B) Schematic of experimental design. Primary human choroid plexus epithelial (HCPEpiC) and endothelial (HCPEC) cells were exposed for 72 h to artificial CSF, non-inflammatory CSF, inflammatory CSF, inflammatory CSF with receptor-specific inhibition (EGFR or VEGFR inhibitors), or non-inflammatory CSF supplemented with recombinant growth factors. (C and E) Representative immunofluorescence images of HCPEpiC (C) and HCPEC (E) stained for proliferating cell nuclear antigen (PCNA) and DAPI following 72-h exposure to experimental conditions. Scale bars, 50 μm. (D) Quantification of PCNA-positive nuclei expressed as percentage of total DAPI-positive cells in epithelial cultures. Data represent mean ± SD from four independent biological experiments, each containing three technical replicate wells (*n* = 4 biological replicates; three technical replicate wells per experiment). Statistical analysis was performed using one-way ANOVA with Tukey post hoc test. Statistical significance: ∗*p* < 0.05, ∗∗*p* < 0.01, ∗∗∗*p* < 0.001, ∗∗∗∗*p* < 0.0001. (F) Quantification of PCNA-positive nuclei in endothelial cultures. Data represent mean + SD from four independent biological experiments, each containing three technical replicate wells (*n* = 4 biological replicates; three technical replicate wells per experiment). Because variance heterogeneity was detected, statistical analysis was performed using Welch’s ANOVA with Games-Howell post hoc testing. ∗*p* < 0.05, ∗∗∗*p* < 0.001, ∗∗∗∗*p* < 0.0001.

These analyses confirm enrichment of inflammatory-associated growth factors—most consistently VEGF—in IVH and TBI CSF relative to SMA controls and support the use of TBI-derived CSF as a growth factor-enriched inflammatory exposure for mechanistic assays.^6^^,^^8^^,^^9^^,^^10^

### Inflammatory CSF induces proliferation in primary human choroid plexus epithelial cells

Quantitative analysis of proliferative responses in primary human ChP epithelial cells demonstrated a significant effect of exposure condition on the proportion of PCNA-positive cells (one-way ANOVA, F(4,15) = 30.81, *p* < 0.0001; R^2^ = 0.8915) (Figures 1C and 1D). Variance assumptions were satisfied (Brown-Forsythe *p* = 0.61; Bartlett’s *p* = 0.41).

Mean epithelial proliferation was 23.7% under artificial CSF control conditions and 28.1% with low-inflammatory SMA CSF. Inflammatory TBI CSF increased proliferation to 42.1%. EGFR inhibition numerically reduced inflammatory CSF-induced proliferation to 27.7%. Supplementation with recombinant EGF increased proliferation to 60.0% when added to artificial CSF and to 71.7% when added to low-inflammatory SMA CSF.

Tukey’s multiple-comparisons testing demonstrated that inflammatory TBI CSF induced significantly greater epithelial proliferation than artificial CSF (mean difference 18.4 percentage points; adjusted *p* = 0.017). The difference relative to low-inflammatory SMA CSF did not reach statistical significance after adjustment (mean difference 14.0 percentage points; adjusted *p* = 0.086). EGFR inhibition produced a numerical reduction relative to inflammatory TBI CSF that did not reach statistical significance (mean difference 14.4 percentage points; adjusted *p* = 0.075); proliferation following EGFR inhibition was not significantly different from artificial CSF control conditions. Recombinant EGF supplementation significantly increased epithelial proliferation from both artificial CSF and low-inflammatory SMA CSF baselines (both adjusted *p* < 0.0001), as well as relative to inflammatory TBI CSF (adjusted *p* < 0.001) (Figures 1C and 1D; Table S1).

These findings demonstrate that inflammatory CSF enhances epithelial proliferative activity and that recombinant EGF is sufficient to induce a robust proliferative response above both artificial CSF and low-inflammatory SMA CSF baselines. Because EGFR inhibition did not reach statistical significance in the epithelial assay (adjusted *p* = 0.075), EGFR pathway dependence is not established by the present experiment.

### Inflammatory CSF promotes proliferation in primary human choroid plexus endothelial cells

Primary human ChP endothelial cells demonstrated a condition-dependent proliferative response. Because variance heterogeneity was detected across groups (Brown–Forsythe ANOVA F(4,10.34) = 20.42, *p* < 0.0001), Welch’s ANOVA was performed to account for unequal variances. Welch’s test confirmed a significant overall effect of exposure condition on endothelial proliferation (W(4,7.093) = 16.44, *p* = 0.0011).

Mean proliferation values were 44.26% under control conditions, 53.68% with SMA CSF, 77.22% with inflammatory TBI CSF, 37.56% with TBI CSF plus VEGFR inhibition, and 69.46% with recombinant VEGF supplementation (Figures 1E and 1F; S2).

Post hoc comparisons were conducted using the Games-Howell test. Inflammatory TBI CSF induced significantly greater endothelial proliferation compared with control conditions (mean difference 32.96%, adjusted *p* = 0.0155). Proliferation in the TBI group also exceeded SMA CSF, although this difference did not reach statistical significance after adjustment (mean difference 23.54%, adjusted *p* = 0.0684). VEGFR inhibition significantly reduced proliferation relative to TBI CSF alone (mean difference 39.66%, adjusted *p* = 0.0069), restoring levels toward baseline. Recombinant VEGF significantly increased proliferation compared with control (mean difference 25.20%, adjusted *p* = 0.0161) and SMA CSF (mean difference 15.78%, adjusted *p* = 0.0078).

Together, these findings indicate that inflammatory CSF stimulates proliferation in ChP endothelial cells and that this response is modulated by VEGF pathway activity, consistent with a growth factor-dependent mechanism.

### Choroid plexus thickness increases with clinical progression to CSF diversion

Quantitative analysis of T2-weighted MRI scans demonstrated progressive enlargement of the ChP across clinical cohorts (Figure 2; Table S3), with demographic characteristics summarized in Table 1.

**Figure 2:**
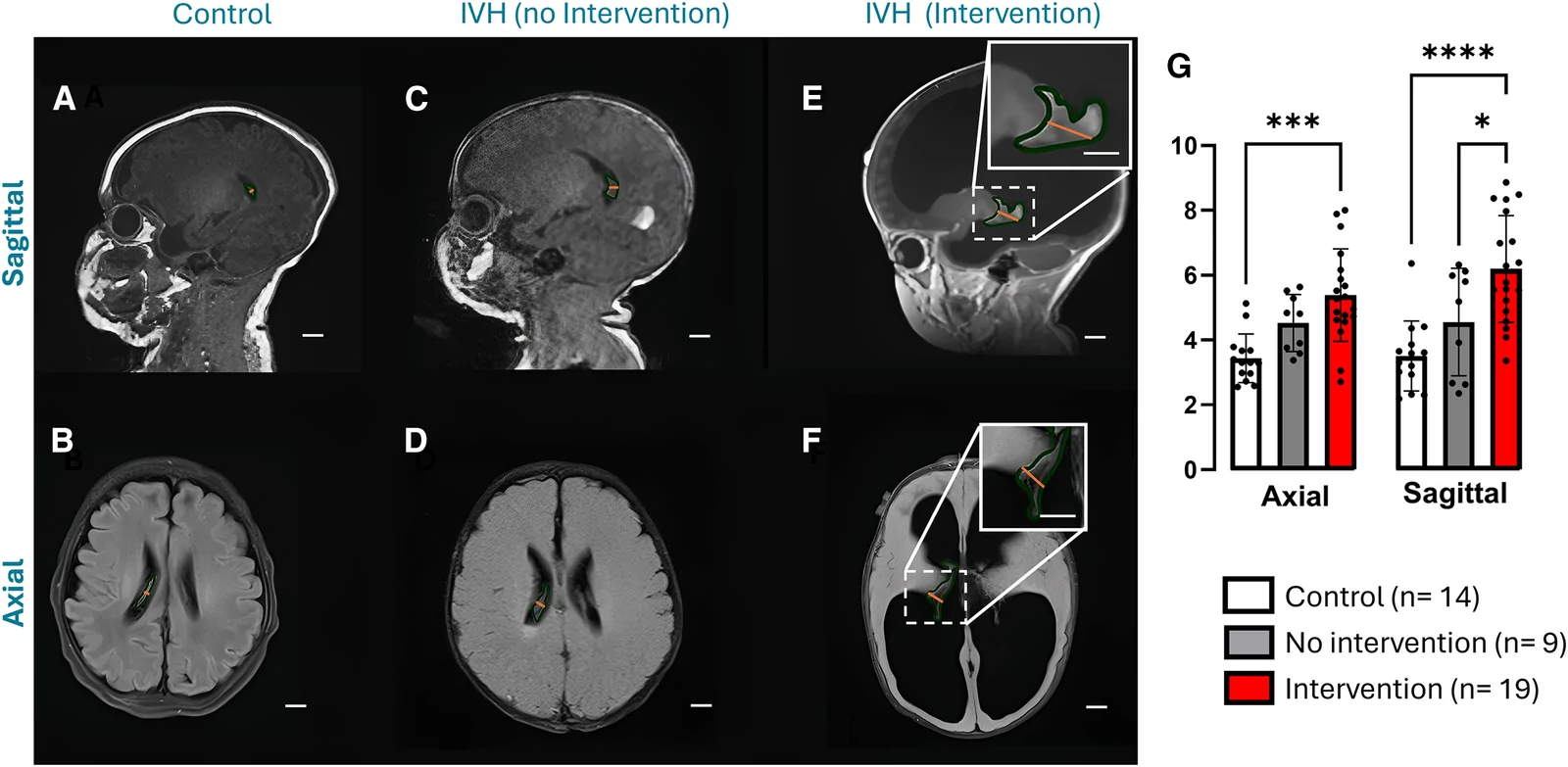
Choroid plexus thickness increases with clinical progression to CSF diversion in preterm infants with intraventricular hemorrhage (A and B) Representative mid-sagittal (A) and axial (B) T2-weighted MRI images from control infants evaluated for suspected hypoxic–ischemic encephalopathy and demonstrating no structural abnormalities. Choroid plexus (ChP) thickness was measured at comparable postmenstrual ages. (C and D) Representative sagittal (C) and axial (D) MRI images from preterm infants with intraventricular hemorrhage (IVH) managed without surgical intervention. (E and F) Representative sagittal (E) and axial (F) MRI images from preterm infants with IVH who subsequently required cerebrospinal fluid (CSF) diversion for hydrocephalus. ChP thickness was measured in both sagittal and axial planes using standardized neuroimaging criteria.^23^^,^^24^ Scale bars, 1 cm. (G) Quantification of ChP thickness across clinical groups. Sagittal ChP thickness measured 3.51 mm in controls (*n* = 14), 4.55 mm in IVH without surgical intervention (*n* = 9), and 6.19 mm in IVH requiring CSF diversion (*n* = 19). Axial ChP thickness measured 3.44 mm, 4.53 mm, and 5.39 mm, respectively. Individual data points and group means ± SD are shown. Statistical analysis was performed using one-way ANOVA with Tukey post hoc testing. Significant differences were observed between the intervention and control groups in both planes (axial: *p* < 0.0001; sagittal: *p* < 0.0001), and between IVH without surgery and intervention groups in the sagittal plane (*p* = 0.025).

**Table 1:** Clinical and imaging characteristics of study cohorts

Variable	Control (*n* = 14)	IVH No surgery (*n* = 9)	IVH + intervention (*n* = 19)
GA at birth (weeks)	37.9	31.72	27.31
PMA at imaging (weeks)	38.96	36.20	37.26
Axial thickness (mm)	3.44	4.53	5.39
Sagittal thickness (mm)	3.51	4.55	6.19

Control infants were born near term and imaged at a mean PMA comparable to the IVH cohorts. Infants requiring CSF diversion were the most premature at birth; however, postmenstrual age at imaging did not substantially differ across groups. Axial and sagittal ChP thickness increased progressively across clinical categories.

In axial planes, mean ChP thickness measured 3.44 mm in control infants (*n* = 14), 4.53 mm in infants with IVH managed without surgical intervention (*n* = 9), and 5.39 mm in infants who progressed to CSF diversion (*n* = 19). One-way ANOVA revealed a significant effect of clinical group (F(2,39) = 11.95, *p* < 0.0001; η^2^ = 0.38), indicating that clinical category accounted for 38% of the observed variance. Post hoc testing demonstrated significantly greater axial thickness in the intervention cohort compared with controls (mean difference 1.95 mm, 95% CI 0.98–2.92, adjusted *p* < 0.0001). The IVH without surgery group did not differ significantly from either controls or the intervention group. Brown-Forsythe testing did not detect variance heterogeneity (*p* = 0.13), supporting the robustness of the model.

Sagittal measurements demonstrated a similar but more pronounced stepwise pattern. Mean sagittal ChP thickness was 3.51 mm in controls, 4.55 mm in IVH without surgical intervention, and 6.19 mm in infants requiring CSF diversion. Group differences were significant (F(2,39) = 13.56, *p* < 0.0001; η^2^ = 0.41), with clinical category explaining 41% of variance. Post hoc analysis confirmed significantly greater sagittal thickness in the intervention group compared with controls (mean difference 2.68 mm, 95% CI 1.41–3.96, adjusted *p* < 0.0001), and a significant difference between IVH without surgery and intervention cohorts (adjusted *p* = 0.025), whereas controls and IVH without surgery did not differ.

To assess whether these differences reflected maturational stage rather than disease trajectory, multivariable linear regression analyses were performed adjusting for gestational age at birth and postmenstrual age (PMA) at imaging. For axial thickness, the model was significant (F(3,38) = 8.05, *p* = 0.0001; R^2^ = 0.42), and clinical category remained independently associated with ChP thickness (β = 1.15 mm per category increase, 95% CI 0.53–1.76, *p* = 0.0005), whereas gestational age at birth and PMA were not significant predictors. Similar results were observed for sagittal thickness (F(3,38) = 9.05, *p* = 0.0001; R^2^ = 0.42), with clinical category independently associated (β = 1.10 mm, 95% CI 0.28–1.91, *p* = 0.0098) and no significant contribution from gestational age or PMA. Residual diagnostics confirmed normal distribution of residuals and absence of problematic multicollinearity.

To determine whether ChP enlargement simply reflected ventricular dilation rather than an independent structural process, multivariable linear regression analyses were performed incorporating ventricular size (FOHR) as a covariate. After adjustment for FOHR, clinical category remained independently associated with axial ChP thickness (β = 0.98 mm per category increase, 95% CI 0.27–1.69, *p* = 0.0078). In contrast, FOHR was not independently associated with axial thickness in the adjusted model (β = −0.06 mm, *p* = 0.98) (Table 2).

**Table 2:** Multivariable linear regression analysis of choroid plexus thickness

Dependent variable	Predictor	β (mm)	95% CI	*p* value
Model A: Primary model (clinical category + FOHR)				
Axial thickness	clinical category	0.98	0.27 to 1.69	0.0078
	FOHR	−0.06	−4.19 to 4.08	0.978
	model R^2^	0.38	–	–
	N	42	–	–
Sagittal thickness	clinical category	0.63	−0.25 to 1.51	0.158
	FOHR	5.16	−0.01 to 10.33	0.0506
	Model R^2^	0.46	–	–
	N	42	–	–
Model B: Sensitivity analysis (clinical category + FOHR + IVH grade)				
Axial thickness	clinical category	0.95	0.11 to 1.80	0.028
	FOHR	−0.09	−4.51 to 4.33	0.969
	IVH grade	0.03	−0.55 to 0.61	0.923
	model R^2^	0.38	–	–
	N	42	–	–
Sagittal thickness	clinical category	1.19	0.19 to 2.19	0.021
	FOHR	6.92	1.70 to 12.15	0.011
	IVH grade	−0.73	−1.41 to −0.04	0.038
	model R^2^	0.52	–	–
	N	42	–	–

Multivariable linear regression models were constructed with choroid plexus thickness as the dependent variable and clinical category, ventricular size (FOHR), and IVH grade as independent variables. Clinical category was coded as an ordinal variable reflecting disease progression (0 = control, 1 = IVH without surgery, 2 = IVH with CSF diversion). IVH grade was coded as an ordinal covariate (0 = no IVH, 1 = grades I-II, 2 = grade III, 3 = grade IV). β values represent change in thickness (mm) per unit increase in predictor. Model A presents the primary model (clinical category + FOHR). Model B presents a sensitivity analysis adding IVH grade as an additional covariate; clinical category remained independently associated with axial ChP thickness after adjustment for IVH grade (β = 0.95 mm, *p* = 0.028), whereas IVH grade itself was not independently associated with axial thickness (*p* = 0.923), confirming that ChP enlargement reflects clinical progression trajectory rather than hemorrhage severity grade alone. FOHR = frontal and occipital horn ratio.

After adjustment for FOHR and IVH grade, clinical category remained independently associated with axial ChP thickness (β = 0.95 mm per category increase, 95% CI 0.11–1.80, *p* = 0.028) and sagittal ChP thickness (β = 1.19 mm per category increase, 95% CI 0.19–2.19, *p* = 0.021). IVH grade was not independently associated with axial thickness (β = 0.03 mm, 95% CI −0.55 to 0.61, *p* = 0.923) but was inversely associated with sagittal thickness (β = −0.73 mm, 95% CI −1.41 to −0.04, *p* = 0.038). These findings confirm that ChP enlargement is associated with clinical progression trajectory independent of hemorrhage severity grade, and that IVH grade has an independent inverse relationship with sagittal ChP thickness after full covariate adjustment.

Although infants requiring CSF diversion were more premature at birth, PMA at imaging was comparable across cohorts. The persistence of the clinical group effect after adjustment for developmental age and ventricular size indicates that ChP enlargement is associated with clinical progression rather than maturational timing or ventricular dilation alone. Together, these findings demonstrate a graded increase in ChP thickness from control infants to IVH without surgical intervention to IVH requiring CSF diversion, independent of developmental stage and ventricular size at imaging.

## Discussion

### Inflammation intersects with developmental immaturity to drive choroid plexus remodeling in preterm hydrocephalus

Acquired hydrocephalus in premature infants most commonly follows intraventricular hemorrhage (IVH) or central nervous system infection.^1^^,^^2^ Although etiologically distinct, both conditions disproportionately affect very preterm infants,^4^^,^^5^^,^^6^ suggesting that developmental state modulates disease expression. The present study integrates MRI-based structural quantification, inflammatory CSF profiling, and primary human ChP cell experiments to support a framework in which inflammatory exposure intersects with developmental immaturity to promote ChP remodeling.

Quantitative MRI analysis demonstrated a progressive increase in ChP thickness across clinical cohorts. Axial thickness increased from 3.44 mm in controls to 4.53 mm in IVH without surgical intervention and 5.39 mm in infants requiring CSF diversion. Sagittal measurements showed a similar stepwise pattern. Importantly, postmenstrual age (PMA) at imaging was comparable across groups, indicating that these structural differences are unlikely to be explained by maturational stage at scan alone.

Multivariable regression analyses confirmed that ChP enlargement was independently associated with clinical progression after adjustment for ventricular size, and that this association persisted after incorporating IVH grade as an additional covariate—indicating that structural remodeling reflects disease trajectory rather than hemorrhage severity grade or developmental immaturity per se. These findings strengthen the interpretation that ChP expansion represents a disease-associated structural response rather than an epiphenomenon of ventricular dilation or prematurity (Table 2).

Infants requiring CSF diversion were born at substantially earlier gestational ages yet were imaged at similar PMA. This dissociation between birth maturity and imaging age is consistent with the possibility that inflammatory exposure occurring within an immature developmental landscape amplifies growth-responsive programs within the ChP.

### Developmental context and inflammatory responsiveness

During late gestation, the ChP retains proliferative capacity and transcriptional plasticity.^20^^,^^36^ Transcriptomic analyses demonstrate dynamic regulation of proliferative and barrier-related programs within the immature ChP.^37^ Growth-factor receptor expression, including EGFR and VEGFR, remains elevated during approximately 24–28 weeks of gestation,^20^^,^^21^ after which these programs attenuate. Prematurity therefore preserves a tissue state that remains responsive to mitogenic signaling.

Preterm infants who progress to hydrocephalus following IVH exhibit elevated CSF concentrations of inflammatory cytokines, including IL-6 and TNF-α, with variable enrichment of VEGF- and EGF-related mediators.^6^^,^^8^^,^^11^^,^^12^ Although neonatal datasets in PIH are more limited,^6^ experimental and pediatric studies indicate similar VEGF- and EGF-linked inflammatory cascades following CNS infection.^13^ Despite distinct initiating insults, these data converge on shared inflammatory growth-factor pathways within the immature ChP. Given this convergence, a growth factor-enriched inflammatory CSF exposure provides a tractable model to test whether such signals are sufficient to activate proliferative programs in human ChP cells.

*In vitro*, exposure of primary human ChP epithelial cells to growth factor-enriched inflammatory CSF increased PCNA-positive cells from approximately 24% under artificial CSF conditions to 42% under inflammatory exposure. In endothelial cells, the corresponding proportions increased from approximately 44%–78%. These proliferative responses were attenuated by EGFR and VEGFR inhibition, supporting receptor-dependent pathway involvement. Together with the recombinant growth-factor experiments, these findings support growth-factor pathway involvement, with stronger pharmacologic evidence for VEGFR than for EGFR dependence.

Importantly, these *in vitro* experiments were designed to test growth factor–dependent proliferative responsiveness and are not intended to model the full etiologic complexity of IVH-derived CSF. The present findings therefore establish biological plausibility and mechanistic sufficiency but do not demonstrate direct causality between inflammatory exposure and MRI-detected ChP enlargement.

When considered alongside the imaging data, these findings support a working framework in which inflammatory signaling intersects with retained developmental competence to promote ChP structural remodeling. Figure 3 integrates these observations into a hypothesis-generating model linking growth factor-enriched inflammatory exposure to proliferative activation and ChP enlargement. Independent published datasets further support the biological plausibility of this framework, including increased Mki67 expression in IVH rat ChP tissue from public RNA-seq data (GSE192946),^11^ elevated inflammatory cytokines during the acute PHH phase (Habiyaremye et al., 2017),^8^ and dynamic changes in the ChP-derived secretory protein transthyretin (TTR) across the PHH treatment course reported by Morales et al. (2012).^38^ Together, these orthogonal datasets support a temporally dynamic remodeling model in which acute inflammatory signaling is associated with proliferative activation and altered epithelial identity, whereas later post-inflammatory stages may involve persistence of a structurally remodeled ChP state with altered constitutive secretory marker profiles (see Figure S1).

**Figure 3:**
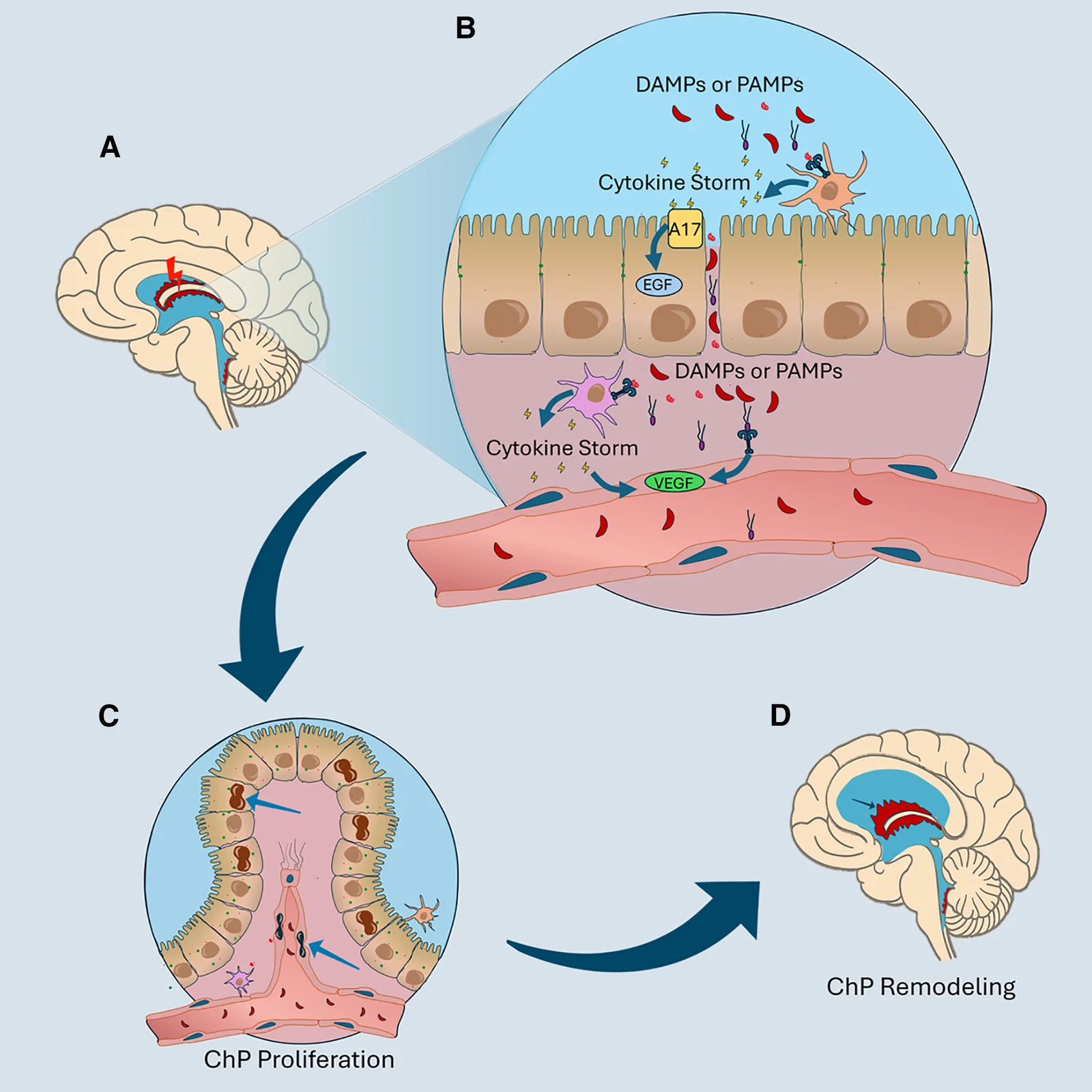
Hypothesis-generating model of inflammation-associated choroid plexus remodeling in preterm acquired hydrocephalus (A) During late gestation, the preterm choroid plexus (ChP) remains developmentally immature and retains proliferative capacity within both epithelial and vascular compartments. Following intraventricular hemorrhage (IVH) or infection, blood-derived danger-associated molecular patterns (DAMPs) or pathogen-associated molecular patterns (PAMPs) enter the ventricular system and contact the ChP surface. (B) Activation of epiplexus and stromal macrophages is associated with local cytokine production (including TNF-α, IL-1β, and IL-6) and enrichment of growth-factor signaling pathways, including EGF- and VEGF-family ligands. In the immature ChP, sustained exposure to growth-factor-rich inflammatory signaling may engage epithelial and endothelial proliferative programs, increasing epithelial surface area and vascular complexity. (C) During periods of developmental immaturity, inflammatory growth-factor signaling may prolong normally time-limited developmental growth programs, resulting in structural expansion of the ChP that persists beyond the acute inflammatory phase. (D) Persistent structural enlargement of the choroid plexus following inflammatory remodeling. CSF secretion rates were not directly measured in this study. See Figure S1 for independent published evidence supporting each component of the model.

### Clinical and translational significance of ChP remodeling

Whether ChP enlargement directly contributes to cerebrospinal fluid (CSF) dysregulation remains unresolved. CSF production is influenced by epithelial surface area, metabolic activity, and growth factor-regulated transport pathways.^39^^,^^40^^,^^41^^,^^42^ However, the present study quantifies structural changes and proliferative responses without directly measuring CSF secretion or transporter activity *in vivo*. Accordingly, ChP enlargement observed on MRI should not be interpreted as evidence of CSF hyperproduction. Therapeutic modulation of glymphatic-meningeal lymphatic drainage has also been identified as a tractable non-surgical strategy in IVH-associated hydrocephalus.^19^

It is biologically plausible that sustained ChP expansion or activation could influence CSF balance in susceptible infants, particularly given prior demonstrations of inflammation-dependent choroidal hypersecretion.^39^^,^^42^^,^^43^ Nonetheless, hydrocephalus is multifactorial. Impaired resorption, arachnoid scarring, altered compliance, and CSF pathway obstruction remain central contributors.^44^^,^^45^^,^^46^ ChP remodeling should therefore be interpreted as one plausible axis within a broader pathophysiologic network.

The observed stepwise differences in ChP thickness suggest that structural remodeling may reflect the combined burden of inflammatory exposure and developmental susceptibility. If validated prospectively, ChP thickness could potentially serve as an adjunct imaging biomarker complementing ventricular indices for risk stratification and disease progression assessment in infants with IVH. Longitudinal studies will be required to determine predictive utility, temporal persistence, and relationship to long-term hydrocephalus outcomes.

Developmental timing may also influence therapeutic opportunity. Should future work confirm that inflammatory signaling during prematurity contributes to sustained ChP remodeling, modulation of growth factor-related pathways within defined maturational windows could represent a testable intervention strategy.

### A structured and testable interaction model in IVH

The data support a developmental susceptibility framework in which the immature ChP retains heightened responsiveness to inflammatory growth-factor signaling.^21^^,^^36^^,^^37^^,^^47^ In our clinical cohort, inflammatory CSF containing elevated EGF and VEGF (Figure 1A) activated EGFR- and VEGFR-dependent proliferative programs in primary human ChP epithelial and endothelial cells (Figures 1B–1D), and this response was associated with measurable ChP enlargement across clinical severity groups on MRI (Figure 2). Independent published datasets further support the biological relevance of this remodeling response in IVH. Reanalysis of GEO dataset GSE192946^11^ demonstrated a 12.7-fold elevation of Mki67 transcripts in IVH rat ChP compared with controls (log2FC = 3.66, *p* = 0.019; Figure S1, panel A), while Ye et al.^22^ showed that hemoglobin—a primary IVH-associated injury mediator—directly induces ChP epithelial proliferation by 37%–65% above control levels.^25^^,^^48^^,^^49^^,^^50^ Together, these findings support a model in which inflammatory exposure during developmental vulnerability promotes proliferative ChP remodeling.

The data also support a testable post-inflammatory remodeling hypothesis. Habiyaremye et al.^8^ demonstrated that IL-1α, IL-6, TNF-α, CCL-19, and CXCL-10 are significantly elevated in acute PHH CSF compared with age-matched controls, but decline with treatment (all *p* < 0.05; Figure S1, B), suggesting temporal separation between the acute inflammatory phase and later structural consequences. Consistent with this transition, Morales et al.^38^ reported that transthyretin (TTR), a constitutive ChP-derived secretory protein, is markedly suppressed during acute PHH (19.8-fold reduction versus controls, *p* < 0.0001) and subsequently increases during treatment progression (fold change 1.13×, *p* = 0.001; Figure S1, C). Together, these observations are compatible with a temporally dynamic remodeling process in which acute inflammatory injury is associated with proliferative activation and altered epithelial identity, whereas later stages may involve persistence of a structurally remodeled ChP state with altered constitutive secretory marker profiles. CSF secretion rates were not directly measured in this study, and the proposed relationship between ChP remodeling and altered secretory capacity therefore remains a testable hypothesis rather than a demonstrated functional outcome.

By framing hydrocephalus progression as the convergence of inflammatory exposure and developmental timing, this work highlights a mechanistically testable axis within a multifactorial disease process. Further longitudinal and functional studies will be required to determine whether structural remodeling contributes to persistent CSF dysregulation and whether this pathway can be therapeutically modulated.

The present study identifies an association between inflammatory CSF exposure and ChP enlargement in preterm infants and demonstrates growth factor-dependent proliferative responses in primary human ChP cells. These findings are consistent with an interaction between inflammatory signaling and retained developmental growth-factor responsiveness in the immature ChP.

### Limitations of the study

Several limitations should be considered. First, although growth factor-dependent proliferative responses were demonstrated *in vitro* and structural enlargement was identified on MRI, CSF secretion, and transporter activity were not directly measured. ChP thickness therefore should not be interpreted as evidence of CSF hyperproduction. Second, inflammatory CSF used for mechanistic assays included samples derived from TBI. Although TBI and IVH CSF shared growth factor enrichment in our cohort, the *in vitro* experiments model exposure to growth factor-enriched inflammatory CSF rather than IVH-specific neonatal CSF composition. These findings therefore demonstrate mechanistic sufficiency rather than IVH-specific causality. Third, although EGFR- and VEGFR-associated proliferative responses were identified using pharmacologic inhibition and recombinant growth factor exposure, downstream signaling intermediates such as ERK1/2 or AKT phosphorylation were not directly evaluated. Additional studies will therefore be required to define pathway specificity and downstream signaling mechanisms in greater detail. Finally, the cross-sectional imaging design limits inference regarding the temporal persistence of ChP enlargement. Longitudinal studies will be required to determine whether structural changes persist beyond the acute inflammatory phase and whether they correlate with long-term CSF dynamics. Additionally, proliferative responses were quantified using PCNA immunolabeling, which is a well-established marker of active DNA synthesis but is also expressed during DNA repair and can persist in post-mitotic cells, potentially leading to overestimation of the true cycling fraction relative to Ki67-based quantification. However, mechanistic concordance between our *in vitro* PCNA findings and the *in vivo* context is supported by independent transcriptomic evidence: reanalysis of GEO: GSE192946 (Robert et al.,)^11^ demonstrated 12.7-fold elevation of Mki67—a cell-cycle-associated marker with greater specificity for actively cycling cells than PCNA—in IVH rat ChP versus controls (log2FC = 3.66, *p* = 0.019; Figure S1A). Although this transcriptomic result does not directly validate PCNA specificity in our cultured cells, the concordant elevation of Mki67 provides orthogonal support for a genuine proliferative response in IVH-associated ChP tissue.

## Resource availability

### Lead contact

Correspondence and requests for materials should be addressed to Leandro Castañeyra-Ruiz, email: leandro.castaneyra.ruiz@choc.org.

### Materials availability

This study did not generate new unique reagents. All primary human ChP epithelial and endothelial cells, media, antibodies, inhibitors, and recombinant proteins used in this study were commercially available. Clinical CSF samples and MRI data cannot be deposited publicly due to patient privacy protections but can be shared in de-identified form upon reasonable request to the lead contact and with appropriate institutional approvals.

### Data and code availability

• De-identified MRI measurements and CSF cytokine datasets are available upon reasonable request to the lead contact, subject to institutional data-sharing policies and patient privacy protections (IRB #2308121). Quantitative values underlying all figures and statistical analyses are provided in Tables S1–S4. Bulk RNA-seq data reanalyzed in this study are available at GEO: GSE192946 (Robert et al.^11^).
• Python code used for transcriptomic reanalysis of GEO: GSE192946 is available upon reasonable request to the lead contact. All other statistical analyses were performed using GraphPad Prism 9 as described in the STAR Methods.
• All other materials are described in the key resources table and are commercially available. Any additional information required to reanalyze the data reported in this paper is available from the lead contact upon request.

## Acknowledgments

We thank colleagues at CHOC Children’s Research Institute for critical discussions and technical support. We especially thank Dr. Alexandra Latini and Celine Tran.

Funding: This work was supported by the 10.13039/100000002National Institutes of Health (NIH R21NS128707-01A1 to L.C.-R.), the U.S. 10.13039/100000005Department of Defense (HT9425-25-1-0112 to L.C.-R.), and institutional funding from CHOC 10.13039/100011301Children's Research Institute through the 10.13039/100025260CSO Small Grants Program (CSO233).

## Author contributions

L.C.-R. conceived the conceptual framework and wrote the manuscript. G.J. and N.R. contributed experimental data and analysis. B.W.H. and M.M. provided clinical interpretation, conceptual refinement, and critical manuscript revision. All authors reviewed and approved the final manuscript.

## Declaration of interests

The authors declare no competing interests.

## Declaration of generative AI and AI-assisted technologies in the manuscript preparation process

The authors used ChatGPT (OpenAI) to assist with language editing and organization of the manuscript. All AI-assisted content was critically reviewed, verified against primary sources, and revised by the authors. The study design, analysis, interpretation, and conclusions represent the original intellectual work of the authors, who take full responsibility for the content.

## STAR★Methods

### Key resources table

**Table:** 

REAGENT or RESOURCE	SOURCE	IDENTIFIER
**Antibodies**		

Mouse anti-PCNA antibody (1:400)	NOVUS Biologicals	Cat# NB500-106
Alexa Fluor 555–conjugated anti-mouse secondary antibody (1:400)	Thermo Fisher Scientific	Cat# 20C0219
DAPI nuclear counterstain (1:5000)	Abcam	Cat# ab228549

**Biological samples**		

Cerebrospinal fluid — IVH-derived (grade III–IV; *n* = 4)	CHOC Children’s Hospital (IRB #2308121)	N/A
Cerebrospinal fluid — TBI-derived (severe TBI; *n* = 2)	CHOC Children’s Hospital (IRB #2308121)	N/A
Cerebrospinal fluid — Non-inflammatory control (SMA-derived)	CHOC Children’s Hospital (IRB #2308121)	N/A

**Chemicals, peptides, and recombinant proteins**		

Erlotinib hydrochloride (EGFR inhibitor)	Sigma-Aldrich	Cat# CDS022564
VEGFR inhibitor V1	Sigma-Aldrich	Cat# 676495-1 MG
Recombinant human EGF (0.16 nM final concentration)	Thermo Fischer	Cat# PHG0313
Recombinant human VEGF (0.02 nM final concentration)	Gibco	Cat #100-20-1 MG
Artificial cerebrospinal fluid (aCSF)	Ecocyte Bioscience	Cat# LRE-S-LSG-1000–2
DMSO (vehicle control)	Sigma Life Science	Cat# D2438
4% Paraformaldehyde (fixative)	Thermo Scientific	Cat# J61899
Triton X-100 (0.1%; permeabilization)	Sigma	Cat# 9036–19-5
BlockAid Blocking Solution	Thermo Fisher	Cat# B10710
PBS (phosphate-buffered saline)	Gibco	Cat# 14190144
Poly-L-lysine (0.1%; epithelial coating)	ScienCell	Cat# 0413
Bovine plasma fibronectin (endothelial coating, 2 μg/mL)	ScienCell	Cat# 8248
Epithelial cell medium (EpiCM)	ScienCell	Cat# 4101
Epithelial cell growth supplement (EpiCGS)	ScienCell	Cat# 4152
Endothelial cell medium (ECM)	ScienCell	Cat# 1001
Endothelial cell growth supplement (ECGS)	ScienCell	Cat# 1052
Penicillin/streptomycin (P/S)	ScienCell	Cat# 0503
Fetal bovine serum (FBS)	ScienCell	Cat# 0025

**Critical commercial assays**		

Luminex Human Inflammation 48-plex Panel (xMAP magnetic bead multiplex)	Creative Proteomics (Shirley, NY, USA)	Custom 48-plex panel

**Deposited data**		

Bulk RNA-seq data — IVH rat ChP (neonatal rat IVH model vs. controls)	Robert et al.,^11^ Cell 186:764–785	GEO: GSE192946
CSF cytokine trajectory in PHH (PHH-IS vs. PHH-TEA; qualitative integration)	Habiyaremye et al.,^8^ Fluids Barriers CNS 14:35	Published data — no accession
CSF proteomic trajectory in PHH — TTR and PTGDS (iTRAQ; PHH-IS vs. PHH-TEA; qualitative integration)	Morales et al.,^38^ Mol Cell Proteomics 11:M111.011973	Published data — no accession

**Experimental models: organisms/strains**		

Neonatal rat IVH model (lateral ventricle blood injection)	Robert et al.^11^ (GSE192946) — reanalysis of published data	N/A (reanalysis only)

**Experimental models: cell lines**		

Primary human choroid plexus epithelial cells (HCPEpiC; passages 3–5)	ScienCell Research Laboratories	Cat# 1310
Primary human choroid plexus endothelial cells (HCPEC; passages 3–5)	ScienCell Research Laboratories	Cat# 1300

**Recombinant DNA**		

No recombinant DNA constructs were generated in this study	N/A	N/A

**Software and algorithms**		

GraphPad Prism 9 (statistical analysis)	GraphPad Software	https://www.graphpad.com; RRID: SCR_002798
Synapse imaging software (MRI measurement)	Fujifilm Medical Systems	https://www.fujifilm.com/us/en/healthcare/medical-imaging
ImageJ (PCNA/DAPI quantification macro)	NIH	https://imagej.nih.gov/ij/; RRID: SCR_003070
GEOparse v2.0.4 (GEO data download)	Python package	https://pypi.org/project/GEOparse/
Python 3.10 (transcriptomic reanalysis)	Python Software Foundation	https://www.python.org; RRID: SCR_008394
EVOS M5000 fluorescence microscope (image acquisition)	Thermo Fisher Scientific	https://www.thermofisher.com

**Other**		

24-well cell culture plates	Thermo Fisher Scientific	Cat# 142485
EVOS M5000 imaging system with 20× objective	Thermo Fisher Scientific	N/A
Synapse digital caliper tool (integrated in Synapse software)	Fujifilm Medical Systems	N/A

### Experimental model and study participant details

#### Human participants — Clinical imaging cohort

Forty-two preterm and term infants were included in the clinical imaging analysis. Infants were enrolled under IRB approval (CHOC IRB #2308121) and in accordance with the Declaration of Helsinki. Three groups were defined: controls (post-HIE, *n* = 14), IVH without CSF diversion (*n* = 9), and IVH with CSF diversion (*n* = 19). Gestational age at birth, postmenstrual age at imaging, IVH grade, and MRI characteristics were recorded for each subject. Sex and gender data were not systematically collected or analyzed in this study; this represents a limitation and future studies should prospectively report sex and gender to evaluate their potential influence on ChP morphology.

#### Human participants — CSF donors

Cerebrospinal fluid samples were obtained from infants with severe IVH (grade III–IV; *n* = 4), traumatic brain injury (*n* = 2), and spinal muscular atrophy (low-inflammatory comparator; *n* = 7) under the same IRB approval (CHOC IRB #2308121). All samples were de-identified prior to analysis.

#### Primary human cell cultures

Primary human ChP epithelial cells (HCPEpiC; ScienCell, Cat# 1310) and primary human ChP endothelial cells (HCPEC; ScienCell, Cat# 1300) were used at passages 3–5. These are commercially available human primary cells derived from adult donors. Species: Homo sapiens. No genotyping or sex-specific characterization was performed on the commercial cell lots used.

#### Public dataset

Bulk RNA-seq data from neonatal rat ChP tissue (IVH model vs. naive controls) were obtained from a publicly available dataset (GEO: GSE192946; Robert et al., 2023, Cell 186:764–785). Species: Rattus norvegicus. No new animal experiments were performed in this study.

### Method details

#### Study approval and patient selection

Sex and gender data were not systematically collected or analyzed in this study. This represents a limitation; future studies should prospectively report sex and gender to evaluate potential influences on ChP morphology and inflammatory responses.

Control infants were included if they underwent evaluation for hypoxic–ischemic encephalopathy and completed therapeutic hypothermia protocols without evidence of intraventricular hemorrhage, infarction, ischemic injury, or structural abnormalities on MRI.

Infants were identified through an IRB-approved electronic medical record query. Infants with intraventricular hemorrhage (IVH) were stratified based on clinical course into those who progressed to hydrocephalus requiring cerebrospinal fluid (CSF) diversion (intervention group) and those who did not require diversion (no-intervention group). Gestational age at birth, postnatal age at imaging, IVH grade, MRI accession number, and imaging sequence type were recorded.

All human sample collection and imaging analyses were conducted under Institutional Review Board approval (CHOC IRB #2308121) and in accordance with the Declaration of Helsinki and institutional guidelines for research involving human subjects.

#### CSF collection and cytokine profiling

Inflammatory CSF samples were obtained from infants with severe (grade III–IV) intraventricular hemorrhage (IVH; *n* = 4) and from patients with severe traumatic brain injury (TBI; *n* = 2). Low-inflammatory comparator CSF was obtained from patients with spinal muscular atrophy (SMA; *n* = 7). All samples underwent multiplex cytokine profiling and categorized as highly inflammatory or low inflammatory.

CSF was collected under sterile conditions, aliquoted, and stored at −80°C until analysis. Cytokine concentrations were quantified using a Luminex Human Inflammation 48-plex Panel (Creative Proteomics, Shirley, NY, USA) based on xMAP magnetic bead multiplex technology. Levels of VEGF, EGF, IL-6, TNF-α, and related inflammatory mediators were measured in each sample.

For *in vitro* exposure experiments, TBI-derived CSF was selected based on two criteria: (1) VEGF concentrations in TBI and IVH CSF were not significantly different by Welch’s ANOVA with Games–Howell post hoc testing (Figure 1A), confirming shared growth factor enrichment; and (2) IVH CSF volume (*n* = 4 samples) was insufficient to conduct triplicate biological replicates across five experimental conditions.

#### Public transcriptomic reanalysis and external dataset integration

To evaluate whether inflammatory proliferative responses observed *in vitro* were biologically relevant to IVH-associated ChP remodeling *in vivo*, we reanalyzed publicly available bulk RNA-seq data from microdissected lateral ventricle ChP tissue obtained from a neonatal rat IVH model (GEO accession GSE192946; Robert et al.).^11^ Raw count data were downloaded from the Gene Expression Omnibus (GEO; https://www.ncbi.nlm.nih.gov/geo/) using GEOparse v2.0.4 in Python 3.10. The dataset comprised ChP tissue from IVH-treated animals (*n* = 5) and naive controls (*n* = 4), collected at 24 h post-injection. Expression values were log_2_(count +1) transformed. Differential expression between IVH and control samples was assessed using Welch’s *t* test. The primary target gene was Mki67 (Ki67), a validated marker of active cell proliferation. No additional filtering or normalization beyond log transformation was applied. Analysis code is available upon reasonable request to the corresponding author.

In parallel, three published experimental and clinical datasets were integrated qualitatively to contextualize the proposed three-component remodeling framework.

(i) *Hemoglobin-induced ChP proliferation:* Ye et al. (2025, *Biomedicines* 13:1366)^22^ reported dose-dependent induction of ChP epithelial proliferation by hemoglobin — the primary IVH mediator — at concentrations 37–65% above control, providing independent functional validation of Component 2 under IVH-specific conditions.
(ii) *CSF inflammatory trajectory in PHH:* Habiyaremye et al. (2017, *Fluids Barriers CNS* 14:35)^8^ reported multiplex CSF cytokine profiling in preterm infants with PHH at initial reservoir placement (PHH-IS, acute phase) and at term-equivalent age following treatment (PHH-TEA). Cytokine concentrations (IL-1α, IL-6, TNF-α, CCL-19, CXCL-10) were extracted from published figures and used to characterize the temporal inflammatory context of Component 2.
(iii) *CSF secretory protein trajectory in PHH:* Morales et al. (2012, *Mol Cell Proteomics* 11:M111.011973)^38^ performed quantitative proteomic profiling of PHH CSF at PHH-IS and PHH-TEA timepoints using isobaric tagging (iTRAQ). Transthyretin (TTR) and prostaglandin D2 synthase (PTGDS/beta-trace) abundance values were extracted from published Tables II and III respectively. These ChP-specific constitutive secretory proteins served as proxies for ChP epithelial secretory activity and were used to characterize the temporal secretory context of Component 3.

All three datasets were used for qualitative contextualization only. No statistical reanalysis was performed on the Ye et al., Habiyaremye et al., or Morales et al. datasets beyond extraction of published summary statistics.

#### MRI acquisition and choroid plexus quantification frontooccipital-horn-ratio (FOHR) quantification

For the primary analysis, one subject-level ChP thickness measurement was obtained in each imaging plane from the side on which the ChP margins were most clearly visualized. When both sides were clearly visualized, the side providing the most complete delineation of ChP boundaries was selected; bilateral measurements were not averaged. This approach was used to minimize measurement uncertainty caused by ventricular distortion, hemorrhagic products, or incomplete visualization of the contralateral ChP in this clinically complex cohort. To address laterality, left- and right-sided measurements were subsequently obtained where visualization permitted. Bilateral axial measurements were available in 14/14 controls (100%), 6/9 infants with IVH without CSF diversion (67%), and 12/19 infants requiring CSF diversion (63%). Bilateral sagittal measurements were available in 13/14 controls (93%), 6/9 without diversion (67%), and 11/19 with diversion (58%). Each subject contributed one index value to the primary analysis; no differential weighting was applied. In a sensitivity analysis using the larger available axial measurement per subject (or the single measurable side in unilateral cases), group differences remained highly significant (F (2,39) = 14.14, *p* < 0.0001), confirming that the primary finding is robust to laterality and measurement-side selection. All individual left, right, and index measurements are provided in Table S4.

FOHR was calculated on axial T2-weighted images as (FHD + OHD)/(2×BPD), where FHD and OHD were measured as the maximal inner-to-inner diameters of the frontal and occipital horns, and BPD as the biparietal inner table–to–inner table diameter at the same level.^51^^,^^52^

To assess measurement reproducibility, a subset of scans was independently quantified by two trained investigators blinded to clinical group assignment. Inter-rater agreement was evaluated using Bland–Altman analysis and intraclass correlation coefficient (ICC; two-way random-effects model, absolute agreement), presented in Figure S2. For axial measurements, ICC = 0.839, mean bias = +0.03 mm (95% CI −0.38 to +0.44 mm), and 95% limits of agreement (LoA) ranged from −1.55 to +1.61 mm. The observed between-group difference in axial ChP thickness (1.95 mm) represents 2.4× the standard deviation of measurement error (SD = 0.805 mm), confirming that group differences substantially exceed inter-rater variability. For sagittal measurements, ICC = 0.946, mean bias = −0.03 mm, and LoA ranged from −1.31 to +1.25 mm, indicating excellent inter-rater reliability.

All measurements were performed using the integrated digital caliper tool within Synapse and recorded in millimeters. Image reviewers were blinded to clinical group assignment and all datasets were de-identified prior to analysis.

#### Primary human choroid plexus cell culture

Primary human ChP epithelial cells (HCPEpiC; ScienCell, #1310) and primary human ChP endothelial cells (HCPEC; ScienCell, #1300) were cultured according to manufacturer recommendations.

Twenty-four–well plates (Thermo Fisher Scientific, #142485) were coated with either 0.1% poly-L-lysine (ScienCell, #0413) for epithelial cultures or bovine plasma fibronectin (ScienCell, #8248) at 2 μg/cm^2^ for endothelial cultures. Plates were incubated at 37°C in 5% CO_2_ for 2 h and washed twice with phosphate-buffered saline (PBS; Gibco, #14190144) prior to seeding.

HCPEpiC were maintained in epithelial cell medium (EpiCM; ScienCell, #4101) supplemented with epithelial cell growth supplement (EpiCGS; ScienCell, #4152), penicillin/streptomycin (P/S; ScienCell, #0503), and fetal bovine serum (FBS; ScienCell, #0025). HCPEC were maintained in endothelial cell medium (ECM; ScienCell, #1001) supplemented with endothelial cell growth supplement (ECGS; ScienCell, #1052), P/S, and FBS. All experiments were performed using cells at passages 3–5.

Cells were seeded at 2.0 × 10^4^ cells/mL (500 μL per well) and allowed to adhere for 1 h before experimental exposure. Cultures were maintained under standard conditions (37°C, 5% CO_2_) for 72 h. Experiments were performed under subconfluent conditions to preserve proliferative responsiveness. Once cultures reached confluence, proliferation markedly decreased due to contact inhibition, limiting detection of growth factor–dependent effects.

#### CSF exposure and pharmacologic manipulation

After initial adhesion, primary human ChP epithelial (HCPEpiC) and endothelial (HCPEC) cells were exposed for 72 h to experimental media composed primarily of artificial CSF (aCSF; Ecocyte Bioscience, #LRE-S-LSG-1000–2), low-inflammatory CSF (SMA-derived), or high-inflammatory CSF (TBI-derived). All exposure conditions were standardized to contain 0.061% dimethyl sulfoxide (DMSO; Sigma Life Science, #D2438), with phosphate-buffered saline (PBS) adjustment to ensure identical vehicle concentrations across groups.

For receptor inhibition experiments, inflammatory CSF conditions were supplemented with erlotinib hydrochloride (EGFR inhibitor; Sigma-Aldrich, #CDS022564) in epithelial cultures or VEGFR inhibitor V1 (Sigma-Aldrich, #676495-1 MG) in endothelial cultures. Inhibitors were diluted in DMSO and added at the start of exposure to achieve the designated final concentrations, while maintaining constant vehicle levels.

To assess signaling sufficiency, recombinant human EGF was added to epithelial cultures at a final concentration of 0.16 nM, and recombinant human VEGF was added to endothelial cultures at a final concentration of 0.02 nM. Recombinant proteins were diluted in PBS immediately prior to application and introduced at the initiation of the 72-h exposure period.

All experimental exposures were performed under identical incubation conditions (37°C, 5% CO_2_), and media were not refreshed during the exposure interval.

#### Immunocytochemistry

After 72 h exposure, cells were washed twice with PBS and fixed in 4% paraformaldehyde (Thermo Scientific, #J61899) for 15 min at room temperature. Media were not refreshed during the 72-h exposure interval to maintain consistent growth factor concentrations throughout the assay period. To confirm that nutrient depletion did not independently influence proliferation, qualitative cell viability was assessed by DAPI nuclear morphology at 72 h; no difference was observed between CSF-exposed and artificial CSF control conditions (Figure 1).

Cells were permeabilized and blocked using 0.1% Triton X-100 (Sigma, #9036-19-5) in BlockAid Blocking Solution (Thermo Fisher, #B10710).

Proliferating cells were labeled with mouse anti-PCNA antibody (NOVUS Biologicals, #NB500-106; 1:400), followed by Alexa Fluor 555–conjugated anti-mouse secondary antibody (Thermo Fisher Scientific, #20C0219; 1:400). Nuclei were counterstained with DAPI (Abcam, #ab228549; 1:5000).

#### Image acquisition and proliferation quantification

Fluorescent images were acquired using an EVOS M5000 fluorescence microscope with a 20× objective. Three randomly selected non-overlapping fields were imaged per well using identical exposure settings across experimental groups.

DAPI-positive nuclei and PCNA-positive cells were quantified using a standardized ImageJ macro, as previously reported.^53^ Proliferation was expressed as the percentage of PCNA-positive nuclei relative to total DAPI-positive nuclei.

### Quantification and statistical analysis

Four independent biological experiments were performed using separate cell preparations, each containing three technical replicate wells per experimental condition. Proliferation values from the three technical wells were averaged within each experiment to generate a single biological replicate value. Statistical analyses were conducted using the mean values from each of the four independent experiments (*n* = 4 biological replicates).

Statistical analyses were performed using GraphPad Prism 9. Data are presented as mean ± standard deviation (SD). Group comparisons were performed using one-way analysis of variance (ANOVA) followed by Tukey’s post hoc multiple comparisons test. When variance heterogeneity was detected (Brown–Forsythe *p* < 0.05), Welch’s ANOVA with Games–Howell post hoc testing was used to account for unequal variances. Statistical significance was defined as *p* < 0.05. Multivariable linear regression analyses were performed with ChP thickness as the dependent variable. Covariates included clinical category (ordinal: 0 = control, 1 = IVH without surgery, 2 = IVH with CSF diversion), FOHR (continuous), gestational age at birth (continuous, weeks), postmenstrual age at imaging (continuous, weeks), and in sensitivity analyses, IVH grade (ordinal: 0 = no IVH; 1 = grades I–II; 2 = grade III; 3 = grade IV). Model assumptions (linearity, homoscedasticity) were assessed using residual plots.

Exact *n* values, definitions of n, statistical tests, and measures of center and dispersion are provided in the corresponding figure legends, tables, and Results. Statistical significance is denoted as follows: ∗*p* < 0.05, ∗∗*p* < 0.01, ∗∗∗*p* < 0.001, and ∗∗∗∗*p* < 0.0001.

## References

[1] Kahle K.T., Klinge P.M., Koschnitzky J.E., Kulkarni A.V., MacAulay N., Robinson S., Schiff S.J., Strahle J.M. (2024). Paediatric hydrocephalus. Nat. Rev. Dis. Primers.

[2] Limbrick J.D.D., de Vries L.S. (2022).

[3] Yang Y., Wang J., Wang K., Liu Z., Ouyang Q., Su Z., Huang J., Xiao G. (2025). Complex Hydrocephalus in Children: Procedure of Surgical Management. Innovat. J. Pediatr..

[4] Robinson S. (2012). Neonatal posthemorrhagic hydrocephalus from prematurity: pathophysiology and current treatment concepts: a review. J. Neurosurg. Pediatr..

[5] Zhou F., Yang Z., Tang Z., Zhang Y., Wang H., Sun G., Zhang R., Jiang Y., Zhou C., Hou X., Liu L. (2023). Outcomes and prognostic factors of infantile acquired hydrocephalus: a single-center experience. BMC Pediatr..

[6] Garcia-Bonilla M., Yahanda A.T., Isaacs A.M., Baksh B., Akbari S.H.A., Botteron H., Morales D.M., Han R.H., McAllister Ii J.P., Mathur A.M. (2024). Pro-inflammatory cerebrospinal fluid profile of neonates with intraventricular hemorrhage: clinical relevance and contrast with CNS infection. Fluids Barriers CNS.

[7] Wu W., Li Q. (2024). Mechanisms of hydrocephalus after intraventricular haemorrhage: a review. Childs Nerv. Syst..

[8] Habiyaremye G., Morales D.M., Morgan C.D., McAllister J.P., CreveCoeur T.S., Han R.H., Gabir M., Baksh B., Mercer D., Limbrick D.D. (2017). Chemokine and cytokine levels in the lumbar cerebrospinal fluid of preterm infants with post-hemorrhagic hydrocephalus. Fluids Barriers CNS.

[9] Lolansen S.D., Rostgaard N., Oernbo E.K., Juhler M., Simonsen A.H., MacAulay N. (2021). Inflammatory markers in cerebrospinal fluid from patients with hydrocephalus: a systematic literature review. Dis. Markers.

[10] Naureen I., Waheed K.A.I., Rathore A.W., Victor S., Mallucci C., Goodden J.R., Chohan S.N., Miyan J.A. (2014). Fingerprint changes in CSF composition associated with different aetiologies in human neonatal hydrocephalus: inflammatory cytokines. Childs Nerv. Syst..

[11] Robert S.M., Reeves B.C., Kiziltug E., Duy P.Q., Karimy J.K., Mansuri M.S., Marlier A., Allington G., Greenberg A.B.W., DeSpenza T. (2023). The choroid plexus links innate immunity to CSF dysregulation in hydrocephalus. Cell.

[12] Gorelik N., Faingold R., Daneman A., Epelman M. (2016). Intraventricular hemorrhage in term neonates with hypoxic-ischemic encephalopathy: a comparison study between neonates treated with and without hypothermia. Quant. Imag. Med. Surg..

[13] Isaacs A.M., Morton S.U., Movassagh M., Zhang Q., Hehnly C., Zhang L., Morales D.M., Sinnar S.A., Ericson J.E., Mbabazi-Kabachelor E. (2021). Immune activation during Paenibacillus brain infection in African infants with frequent cytomegalovirus co-infection. iScience.

[14] Preissner K.T., Fischer S., Deindl E. (2020). Extracellular RNA as a versatile DAMP and alarm signal that influences leukocyte recruitment in inflammation and infection. Front. Cell Dev. Biol..

[15] Castañeyra-Ruiz L., Latini A., Hanak B.W., Muhonen M. (2026). Beyond structural fragility in germinal matrix hemorrhage: a protease-threshold model of developmental cerebral microvascular failure. J. Cereb. Blood Flow Metab..

[16] Castañeyra-Ruiz L., Garcia-Bonilla M., Isaacs A.M., Morales D.M., Otun A., Michenkova M., Zwick S.H., Wardenburg J.B., Paulson J.N., Castañeyra-Perdomo A. (2026). ADAM10-dependent disruption of N-cadherin and ventricular zone in post-hemorrhagic hydrocephalus. Exp. Neurol..

[17] Zhang Q., Niu Y., Li Y., Xia C., Chen Z., Chen Y., Feng H. (2025). Meningeal lymphatic drainage: novel insights into central nervous system disease. Signal Transduct. Target. Ther..

[18] Pinho-Correia L.M., McCullough S.J.C., Ghanizada H., Nedergaard M., Rustenhoven J., Da Mesquita S. (2025). CSF transport at the brain–meningeal border: effects on neurological health and disease. Lancet Neurol..

[19] Yang Y., Ouyang Q., Sun Y., Liang A., Wang J., Wang K., Yuan Y., Liu Z., Huang J., Zhang Z. (2026). Therapeutic Hypothermia Alleviates Hydrocephalus and Neurological Dysfunction Post Intraventricular Hemorrhage by Enhancing Drainage of Glymphatic-Meningeal Lymphatic-Deep Cervical Lymphatic System. CNS Neurosci. Ther..

[20] Ortenlöf N., Vallius S., Karlsson H., Ekström C., Kristiansson A., Holmqvist B., Göransson O., Vaváková M., Rydén M., Carey G. (2023). Characterization of choroid plexus in the preterm rabbit pup following subcutaneous administration of recombinant human IGF-1/IGFBP-3. Fluids Barriers CNS.

[21] Barkho B.Z., Monuki E.S. (2015). Proliferation of cultured mouse choroid plexus epithelial cells. PLoS One.

[22] Ye H., Miao W., Keep R.F., Xiang J. (2025). Growth Factors and the Choroid Plexus: Their Role in Posthemorrhagic Hydrocephalus. Biomedicines.

[23] Madhukar M., Choudhary A.K., Boal D.K., Dias M.S., Iantosca M.R. (2012). Choroid plexus: normal size criteria on neuroimaging. Surg. Radiol. Anat..

[24] Kim H., Lim Y.-M., Kim G., Lee E.J., Lee J.H., Kim H.W., Kim K.K. (2020). Choroid plexus changes on magnetic resonance imaging in multiple sclerosis and neuromyelitis optica spectrum disorder. J. Neurol. Sci..

[25] Fleischer V., Gonzalez-Escamilla G., Ciolac D., Albrecht P., Küry P., Gruchot J., Dietrich M., Hecker C., Müntefering T., Bock S. (2021). Translational value of choroid plexus imaging for tracking neuroinflammation in mice and humans. Proc. Natl. Acad. Sci. USA.

[26] Sun S., Chen Y., Yun Y., Zhao B., Ren Q., Sun X., Meng X., Yan C., Lin P., Liu S. (2024). Elevated peripheral inflammation is associated with choroid plexus enlargement in independent sporadic amyotrophic lateral sclerosis cohorts. Fluids Barriers CNS.

[27] Heep A., Stoffel-Wagner B., Bartmann P., Benseler S., Schaller C., Groneck P., Obladen M., Felderhoff-Mueser U. (2004). Vascular endothelial growth factor and transforming growth factor-β1 are highly expressed in the cerebrospinal fluid of premature infants with posthemorrhagic hydrocephalus. Pediatr. Res..

[28] Shim J.W., Sandlund J., Hameed M.Q., Blazer-Yost B., Zhou F.C., Klagsbrun M., Madsen J.R. (2016). Excess HB-EGF, which promotes VEGF signaling, leads to hydrocephalus. Sci. Rep..

[29] Tito C., Masciarelli S., Colotti G., Fazi F. (2025). EGF receptor in organ development, tissue homeostasis and regeneration. J. Biomed. Sci..

[30] Breier G., Albrecht U., Sterrer S., Risau W. (1992). Expression of vascular endothelial growth factor during embryonic angiogenesis and endothelial cell differentiation. Development.

[31] Clemente L., Bird I.M. (2023). The epidermal growth factor receptor in healthy pregnancy and preeclampsia. J. Mol. Endocrinol..

[32] Potenzieri A., Uccella S., Preiti D., Pisoni M., Rosati S., Lavarello C., Bartolucci M., Debellis D., Catalano F., Petretto A. (2024). Early IGF-1 receptor inhibition in mice mimics preterm human brain disorders and reveals a therapeutic target. Sci. Adv..

[33] Hakonen E., Ustinov J., Mathijs I., Palgi J., Bouwens L., Miettinen P.J., Otonkoski T. (2011). Epidermal growth factor (EGF)-receptor signalling is needed for murine beta cell mass expansion in response to high-fat diet and pregnancy but not after pancreatic duct ligation. Diabetologia.

[34] Gilard V., Chadie A., Ferracci F.-X., Brasseur-Daudruy M., Proust F., Marret S., Curey S. (2018). Post hemorrhagic hydrocephalus and neurodevelopmental outcomes in a context of neonatal intraventricular hemorrhage: an institutional experience in 122 preterm children. BMC Pediatr..

[35] Khalaveh F., Zeiser V., Cho A., Schmelzer S., Reinprecht A., Herta J., Roessler K., Dorfer C. (2023). Predicting the need for cerebrospinal fluid shunt implantation after spontaneous intracerebral hemorrhage: a challenging task. Front. Neurol..

[36] Castañeyra-Ruiz L., González-Marrero I., Hernández-Abad L.G., Carmona-Calero E.M., Meyer G., Castañeyra-Perdomo A. (2016). A distal to proximal gradient of human choroid plexus development, with antagonistic expression of Glut1 and AQP1 in mature cells vs. calbindin and PCNA in proliferative cells. Front. Neuroanat..

[37] Kratzer I., Liddelow S.A., Saunders N.R., Dziegielewska K.M., Strazielle N., Ghersi-Egea J.-F. (2013). Developmental changes in the transcriptome of the rat choroid plexus in relation to neuroprotection. Fluids Barriers CNS.

[38] Morales D.M., Townsend R.R., Malone J.P., Ewersmann C.A., Macy E.M., Inder T.E., Limbrick D.D. (2012). Alterations in protein regulators of neurodevelopment in the cerebrospinal fluid of infants with posthemorrhagic hydrocephalus of prematurity. Mol. Cell. Proteomics.

[39] MacAulay N., Keep R.F., Zeuthen T. (2022). Cerebrospinal fluid production by the choroid plexus: a century of barrier research revisited. Fluids Barriers CNS.

[40] Lolansen S.D., Révész E.O., Andreassen S.N., Trapp M., Salio C., Sassoé-Pognetto M., Andersen J.V., Westi E.W., Toft-Bertelsen T.L., Wardman J.H. (2025). Choroid plexus-mediated CSF secretion remains stable in aging rats via high and age-resistant metabolic activity. Nat. Commun..

[41] Katada S., S Rodrigues K., Nakashima K. (2025). The influence of the choroid plexus on brain function: beyond its role in cerebrospinal fluid production. Inflamm. Regen..

[42] Johnsen L.Ø., Friis K.A., Møller-Madsen M.K., Damkier H.H. (2025). Mechanisms of cerebrospinal fluid secretion by the choroid plexus epithelium: Application to various intracranial pathologies. Clin. Anat..

[43] Karimy J.K., Zhang J., Kurland D.B., Theriault B.C., Duran D., Stokum J.A., Furey C.G., Zhou X., Mansuri M.S., Montejo J. (2017). Inflammation-dependent cerebrospinal fluid hypersecretion by the choroid plexus epithelium in posthemorrhagic hydrocephalus. Nat. Med..

[44] Lolansen S.D., Rostgaard N., Barbuskaite D., Capion T., Olsen M.H., Norager N.H., Vilhardt F., Andreassen S.N., Toft-Bertelsen T.L., Ye F. (2022). Posthemorrhagic hydrocephalus associates with elevated inflammation and CSF hypersecretion via activation of choroidal transporters. Fluids Barriers CNS.

[45] Bravo M.C., Lubian S., Horsch S., Cabañas F., de Vries L.S., EurUS.Brain group (2024). Neonatal ventriculomegaly: Pathophysiology and management guided with cranial ultrasonography. Dev. Med. Child Neurol..

[46] Hladky S.B., Barrand M.A. (2024). Regulation of brain fluid volumes and pressures: basic principles, intracranial hypertension, ventriculomegaly and hydrocephalus. Fluids Barriers CNS.

[47] Masters H., Wang S., Tu C., Nguyen Q., Sha Y., Karikomi M.K., Fung P.S.R., Tran B., Martel C., Kwang N. (2025). Sequential emergence and contraction of epithelial subtypes in the prenatal human choroid plexus revealed by a stem cell model. Nat. Commun..

[48] Rau A., Gonzalez-Escamilla G., Schroeter N., Othman A., Dressing A., Weiller C., Urbach H., Reisert M., Groppa S., Hosp J.A. (2024). Inflammation-triggered enlargement of choroid plexus in subacute COVID-19 patients with neurological symptoms. Ann. Neurol..

[49] Ricigliano V.A.G., Morena E., Colombi A., Tonietto M., Hamzaoui M., Poirion E., Bottlaender M., Gervais P., Louapre C., Bodini B., Stankoff B. (2021). Choroid plexus enlargement in inflammatory multiple sclerosis: 3.0-T MRI and translocator protein PET evaluation. Radiology.

[50] Krieger B., Bellenberg B., Roenneke A.K., Schneider R., Ladopoulos T., Abbas Z., Rust R., Schmitz-Hübsch T., Chien C., Gold R. (2025). Relevance of choroid plexus volumes in multiple sclerosis. Fluids Barriers CNS.

[51] Helmbold L.J., Kammler G., Regelsberger J., Fritzsche F.S., Emami P., Schüller U., Krajewski K. (2019). Predictive factors associated with ventriculoperitoneal shunting after posterior fossa tumor surgery in children. Childs Nerv. Syst..

[52] O’hayon B., Drake J.M., Ossip M.G., Tuli S., Clarke M. (1998). Frontal and occipital horn ratio: a linear estimate of ventricular size for multiple imaging modalities in pediatric hydrocephalus. Pediatr. Neurosurg..

[53] Castaneyra-Ruiz L., Morales D.M., McAllister J.P., Brody S.L., Isaacs A.M., Strahle J.M., Dahiya S.M., Limbrick D.D. (2018). Blood exposure causes ventricular zone disruption and glial activation in vitro. J. Neuropathol. Exp. Neurol..

