# Supplementary material for: Choroid plexus remodeling associated with inflammatory CSF exposure in preterm acquired hydrocephalus
Source: iScience. 2026 Oct 1;29(10):117759. doi: 10.1016/j.isci.2026.117759 (PMC13657226; doi:10.1016/j.isci.2026.117759)
Supplement: Supplementary file 1 [file mmc1.pdf]

iScience, Volume *29*

Supplemental information

Choroid plexus remodeling associated with inflammatory CSF exposure in preterm acquired hydrocephalus

Gio Jison, Noah Ramos, Fernando Cotrim-Gomes, Seunghyun Lee, Brian W. Hanak, Michael Muhonen, and Leandro Castañeyra-Ruiz

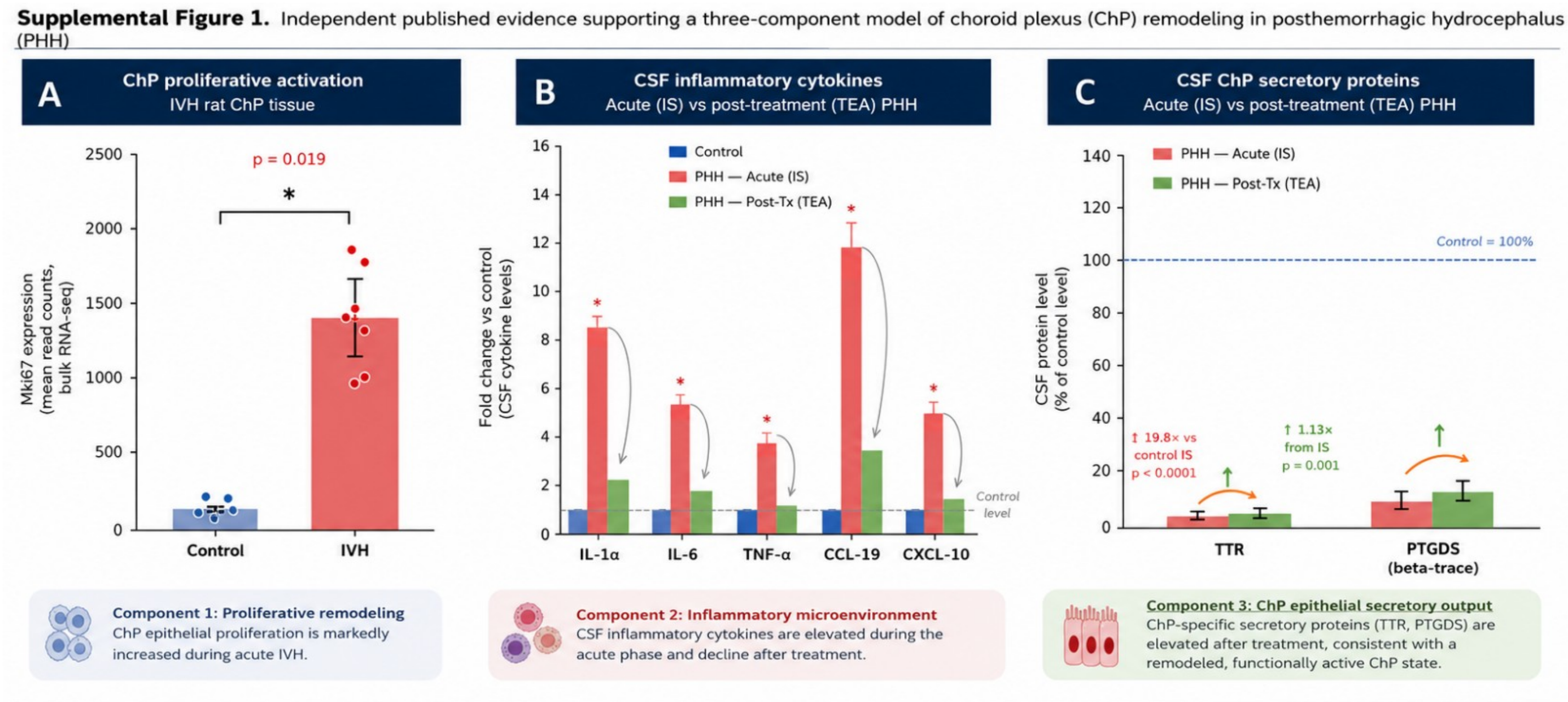

Supplementary Figure 1. Independent published evidence supporting a dynamic model of choroid plexus (ChP) remodeling in posthemorrhagic hydrocephalus (PHH).

(A) Reanalysis of public bulk RNA-seq data from rat IVH ChP tissue (GSE192946; Robert et al., 2023) showing marked elevation of Mki67 transcripts in IVH versus controls, supporting proliferative ChP remodeling in vivo. (B) Published CSF cytokine profiling in PHH infants (Habiyaremye et al., 2017) demonstrating elevated inflammatory cytokines during the acute PHH phase with decline following treatment. (C) Published CSF proteomic data (Morales et al., 2012) showing suppression of the ChP-derived secretory protein transthyretin (TTR) during acute PHH followed by partial recovery during treatment progression. Together, these datasets support a temporally dynamic remodeling model in which acute inflammatory activation is associated with proliferative and epithelial stress responses, whereas later stages may involve persistence of a structurally remodeled ChP state with altered constitutive secretory marker profiles. CSF secretion rates were not directly measured, and the proposed relationship between ChP remodeling and altered secretory capacity remains a testable hypothesis rather than a demonstrated functional outcome.

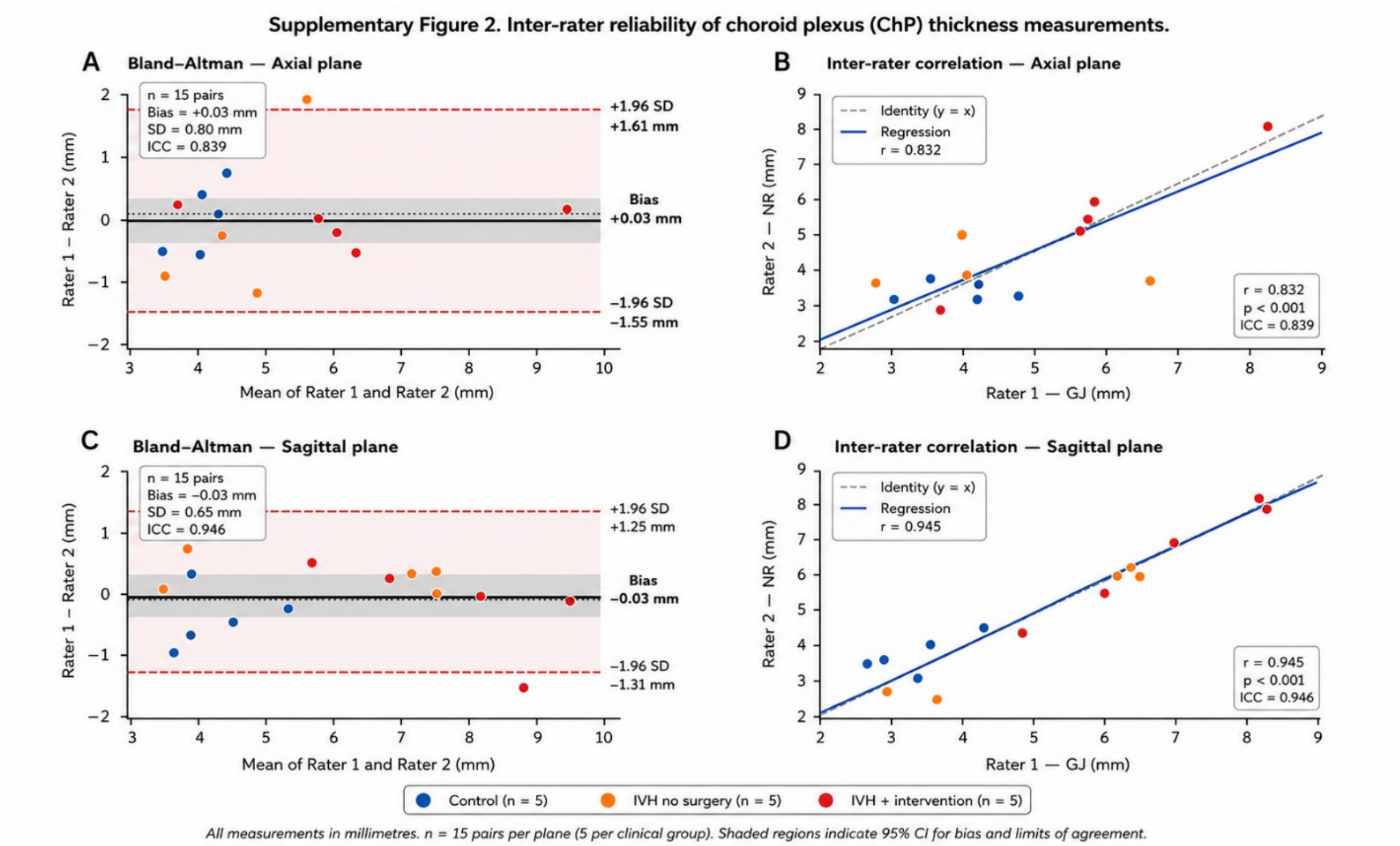

Supplementary Figure 2. Inter-rater reliability of choroid plexus (ChP) thickness measurements. Bland–Altman analysis and inter-rater correlation by imaging plane.

(A) Bland–Altman plot showing agreement between two independent raters for axial ChP thickness measurements (n = 15 pairs). Mean bias = +0.03 mm; 95% limits of agreement (LoA) = −1.55 to +1.61 mm; ICC = 0.839. Shaded regions indicate 95% confidence intervals for the bias and LoA.
(B) Inter-rater correlation for axial ChP thickness measurements. Dashed line indicates identity (y = x); solid line indicates least-squares regression (r = 0.832, $p < 0.001$, ICC = 0.839).
(C) Bland–Altman plot showing agreement between two independent raters for sagittal ChP thickness measurements (n = 15 pairs). Mean bias = −0.03 mm; 95% LoA = −1.31 to +1.25 mm; ICC = 0.946. Shaded regions indicate 95% confidence intervals for the bias and LoA**.**
(D) Inter-rater correlation for sagittal ChP thickness measurements (r = 0.945, $p < 0.001$, ICC = 0.946).
Blue = control, orange = IVH without surgical intervention, red = IVH requiring CSF diversion (n = 5 per group). Measurements were performed independently by both raters.

Supplementary Table 1. HCPEpiC Proliferation Quantification.

Quantification of PCNA-positive nuclei expressed as percentage of total DAPI-positive cells in primary human choroid plexus epithelial (HCPEpiC) cultures following 72-hour exposure to each experimental condition. Data represent mean ± SD from three independent biological experiments, each consisting of four technical replicate wells per condition ($n = 3$ biological replicates). Proliferation index calculated as PCNA-positive cells divided by total DAPI-positive cells. Statistical analysis performed using one-way ANOVA with Tukey's post hoc test. Variance assumptions satisfied (Brown–Forsythe $p = 0.61$; Bartlett's $p = 0.41$). EGFR inh = erlotinib hydrochloride (EGFR inhibitor); aCSF = artificial cerebrospinal fluid; SMA = spinal muscular atrophy-derived CSF (low-inflammatory); TBI = traumatic brain injury-derived CSF (inflammatory).

Condition	N (biological replicates)	Total Cells (DAPI) Mean ± SD	PCNA+ Cells Mean ± SD	Proliferation Index (% PCNA/DAPI) Mean ± SD
**Control (−) — Artificial CSF**	3	189.2 ± 31.1	40.8 ± 9.0	23.7 ± 7.7
**Low-inflammatory CSF (SMA)**	3	191.8 ± 8.2	53.4 ± 20.2	28.1 ± 10.5
**Inflammatory CSF (TBI)**	3	306.9 ± 22.0	126.2 ± 15.7	42.1 ± 7.3
**Inflammatory CSF + EGFR inh (Treated TBI)**	3	136.8 ± 34.4	37.9 ± 11.5	27.7 ± 4.2
**aCSF + Recombinant EGF**	3	254.4 ± 35.3	150.3 ± 8.0	60.0 ± 5.4
**SMA CSF + Recombinant EGF**	3	224.3 ± 34.3	160.2 ± 26.4	71.7 ± 3.4

Statistical comparisons (Tukey's post hoc test): TBI vs. artificial CSF: mean difference 18.4 pp, adjusted $p = 0.017$. TBI vs. SMA CSF: mean difference 14.0 pp, adjusted $p = 0.086$ (ns). EGFR inhibition vs. TBI: mean difference 14.4 pp, adjusted $p = 0.075$ (ns). aCSF + Recombinant EGF vs. artificial CSF: adjusted $p < 0.0001$. aCSF + Recombinant EGF vs. SMA CSF: adjusted $p < 0.0001$. SMA + Recombinant EGF vs. artificial CSF: adjusted $p < 0.0001$. SMA + Recombinant EGF vs. TBI: adjusted $p < 0.001$. ns = not significant; pp = percentage points.

Supplementary Table 2. HCPEC Culture Quantification

Condition	n (biological replicates)	Total Cells (DAPI) Mean ± SD	PCNA+ Cells Mean ± SD	Proliferation Index (% PCNA/DAPI) Mean ± SD
Control (−)	4	59.7 ± 6.2	26.6 ± 6.8	44.2 ± 8.2
SMA	4	64.9 ± 5.5	37.7 ± 3.1	53.7 ± 3.2
TBI	4	64.9 ± 5.5	54.5 ± 7.4	77.7 ± 9.4
Treated TBI	4	65.7 ± 9.0	26.3 ± 5.7	37.6 ± 8.4
aCSF + Recombinant	4	64.4 ± 9.7	42.8 ± 9.7	69.8 ± 10.5
SMA + Recombinant	4	76.1 ± 18.7	64.7 ± 10.0	69.5 ± 4.2

Data represent mean ± SD from four independent biological replicates, each consisting of three technical wells per condition. Proliferation index calculated as PCNA-positive cells divided by total DAPI-positive cells.

Supplementary Table 3. Clinical and Imaging Characteristics of Study Participants

Abbreviations: GA, gestational age at birth; PMA, postmenstrual age at imaging; IVH, intraventricular hemorrhage; HIE, hypoxic-ischemic encephalopathy; ChP, choroid plexus; FOHR, frontal–occipital horn ratio; VAD, ventricular access device; EVD, external ventricular drain.

Controls (Post-HIE; No CSF Diversion)

ID	GA (wks)	PMA (wks)	Etiology	Axial ChP (mm)	Sagittal ChP (mm)	FOHR
C1	37	39	Post-HIE	3.79	6.36	0.3410
C2	36	37	Post-HIE	3.30	2.94	0.2630
C3	36	37	Post-HIE	3.40	3.30	0.2606
C4	36	38	Post-HIE	3.30	3.86	0.2852
C5	37	38	Post-HIE	3.67	4.41	0.3186
C6	39	40	Post-HIE	2.97	2.32	0.2407
C7	38	38.5	Post-HIE	2.59	3.02	0.2617
C8	38	39	Post-HIE	3.29	3.65	0.2599
C9	39	39	Post-HIE	5.13	3.61	0.2254
C10	39	40	Post-HIE	4.75	4.36	0.2579
C11	40	41	Post-HIE	3.68	3.20	0.2918
C12	36	37	Post-HIE	2.55	2.19	0.2418
C13	40	41	Post-	2.99	3.59	0.2759

ID	GA (wks)	PMA (wks)	Etiology	Axial ChP (mm)	Sagittal ChP (mm)	FOHR
			HIE			
C14	40	41	Post-HIE	2.73	2.30	0.2771
**Mean ± SD**	**37.9 ± 1.6**	**39.0 ± 1.5**	**—**	**3.44 ± 0.75**	**3.51 ± 1.08**	**0.272 ± 0.031**

IVH Without CSF Diversion

ID	GA (wks)	PMA (wks)	IVH Grade	Axial ChP (mm)	Sagittal ChP (mm)	FOHR
N1	40	41	pIVH	3.90	2.67	0.3324
N2	35–36	37	pIVH	4.84	6.32	0.3782
N3	40	41	Grade 3	3.59	2.35	0.3709
N4	27	31	Grade 3–4	3.76	5.80	0.5349
N5	29	37	Grade 4	5.29	6.12	0.5480
N6	35	36	Grade 4	5.64	3.92	0.5723
N7	27	30	Grade 4	4.81	5.19	0.4632
N8	25	45	Grade 4	3.38	2.63	0.6351
N9	27	28	pIVH	5.52	5.99	0.4559
**Mean ± SD**	**31.7 ± 6.0**	**36.2 ± 5.7**	—	**4.53 ± 0.86**	**4.55 ± 1.67**	**0.477 ± 0.099**

IVH Requiring CSF Diversion (VAD/Shunt/EVD)

ID	GA (wks)	PMA (wks)	IVH Grade	Diversion Type	Axial ChP (mm)	Sagittal ChP (mm)	FOHR
S1	24–25	36	pIVH	VAD	5.52	4.89	0.3976
S2	36	38	pIVH	Shunt	3.05	5.54	0.6487
S3	29	65	pIVH	Shunt	5.27	8.49	0.6960
S4	27	27	pIVH	Shunt	5.67	4.35	0.4281
S5	22	38	Grade 4	Shunt	4.95	6.99	0.6654
S6	28–29	32	pIVH	VAD	8.00	8.34	0.5558
S7	26	30	pIVH	VAD	5.34	5.57	0.6499
S8	24	34	Grade 4	EVD + Shunt	4.76	5.22	0.4274
S9	28	48	Grade 4	Shunt	4.26	3.36	0.4825
S10	24	44	Grade 4	Shunt	4.73	4.57	0.6332
S11	23	29	pIVH	Shunt	7.50	8.86	0.6101
S12	31	35	Grade 4	Shunt	6.16	5.77	0.4710
S13	24	38	Grade 3	Shunt	4.86	8.37	0.7027
S14	28	32	pIVH	Shunt	2.71	5.55	0.5384
S15	37	37	pIVH	Shunt	7.89	6.29	0.5401
S16	25	41	Grade 4	Shunt	5.88	6.39	0.5232
S17	24	33	Grade 3	VAD	4.62	7.04	0.3923
S18	35	44	IVH	VP Shunt	6.66	7.97	0.6057
S19	24	27	IVH	VP Shunt	4.56	4.09	0.5974
**Mean ± SD**	**26.9 ± 3.9**	**37.0 ± 8.2**	—	—	**5.39 ± 1.43**	**6.19 ± 1.64**	**0.552 ± 0.105**

Supplementary Table 4. Individual choroid plexus thickness measurements from the 42-subject primary analysis cohort.

The primary analysis used one subject-level index ChP thickness measurement per imaging plane (Axial Index; Sagittal Index), corresponding to the best-visualized ChP cross-section at the level of maximal prominence, as reported in Supplementary Table 3. Left (L) and right (R) measurements were subsequently obtained where visualization permitted to address reviewer concerns regarding laterality. Bilateral = both L and R measurements obtainable. Dom = dominant ventricle side (side of greater IVH burden or larger measurement when not lateralized). n/a = not measurable due to ventricular distortion, blood products, or imaging quality. The sensitivity analysis used the larger of L and R (or the available side if unilateral); $F[2,39] = 14.14$, $p < 0.0001$.

ID	Group	GA (wk)	PMA (wk)	Etiology / Diversion	Axial Index (mm)	Axial L (mm)	Axial R (mm)	Axial Larger side	Sagittal Index (mm)	Sagittal L (mm)	Sagittal R (mm)	Dominant Ventricle	Axial Bilateral?	Sagittal Bilateral?
C1	Control	37	39	Post-HIE	3.79	4.30	3.79	4.30	6.36	5.30	6.36	Right	Yes	Yes
C2	Control	36	37	Post-HIE	3.30	3.30	3.80	3.80	2.94	2.68	2.94	Neutral	Yes	Yes
C3	Control	36	37	Post-HIE	3.40	3.24	3.40	3.40	3.30	3.30	2.67	Neutral	Yes	Yes
C4	Control	36	38	Post-HIE	3.30	3.30	3.15	3.30	3.86	3.86	3.31	Neutral	Yes	Yes
C5	Control	37	38	Post-HIE	3.67	3.18	3.67	3.67	4.41	4.01	4.41	Neutral	Yes	Yes
C6	Control	39	40	Post-HIE	2.97	2.97	1.88	2.97	2.32	2.32	2.23	Neutral	Yes	Yes
C7	Control	38	38.5	Post-HIE	2.59	3.50	2.59	3.50	3.02	3.02	2.19	Neutral	Yes	Yes
C8	Control	38	39	Post-HIE	3.29	3.04	3.29	3.29	3.65	3.65	n/a	Neutral	Yes	No
C9	Control	39	39	Post-HIE	5.13	5.13	4.57	5.13	3.61	3.54	3.61	Neutral	Yes	Yes
C10	Control	40	41	Post-HIE	4.75	4.75	4.58	4.75	4.36	4.36	4.01	Neutral	Yes	Yes
C11	Control	40	41	Post-HIE	3.68	3.37	3.68	3.68	3.20	3.20	3.37	Neutral	Yes	Yes
C12	Control	36	37	Post-HIE	2.55	2.55	2.15	2.55	2.19	2.19	2.15	Neutral	Yes	Yes
C13	Control	40	41	Post-HIE	2.99	3.24	2.99	3.24	3.59	3.59	2.97	Neutral	Yes	Yes
C14	Control	40	41	Post-HIE	2.73	2.26	2.73	2.73	2.30	2.37	2.30	Neutral	Yes	Yes
—	**IVH — No Surgery**	—	—	—	—	—	—	—	—	—	—	—	—	—
N1	IVH No Surg	40	41	pIVH	3.90	3.60	3.90	3.90	2.67	2.67	2.97	Neutral	Yes	Yes
N2	IVH No Surg	35–36	37	pIVH	4.84	4.84	5.03	5.03	6.32	5.62	6.32	Neutral	Yes	Yes
N3	IVH No Surg	40	41	Grade 3	3.59	4.67	3.59	4.67	2.35	2.35	4.13	Neutral	Yes	Yes
N4	IVH No Surg	27	31	Grade 3–4	3.76	n/a	3.76	3.76	5.80	5.80	4.32	Neutral	No	Yes
N5	IVH No Surg	29	34	Grade 4	5.29	4.94	5.29	5.29	6.12	6.90	6.12	Right	Yes	Yes
N6	IVH No Surg	35	36	Grade 4	5.64	5.64	n/a	5.64	3.92	3.92	n/a	Neutral	No	No
N7	IVH No Surg	27	30	Grade 4	4.81	4.81	n/a	4.81	5.19	5.19	n/a	Right	No	No
N8	IVH No Surg	25	45	Grade 4	3.38	3.11	3.38	3.38	2.63	2.45	2.63	Right	Yes	Yes
N9	IVH No Surg	27	28	pIVH	5.52	5.52	4.58	5.52	5.99	5.99	n/a	Neutral	Yes	No
—	**IVH — CSF Diversion**	—	—	—	—	—	—	—	—	—	—	—	—	—
S1	IVH Diversion	24–25	36	pIVH / VAD	5.52	5.11	5.52	5.52	4.89	4.76	4.89	Right	Yes	Yes
S2	IVH Diversion	36	38	pIVH / Shunt	3.05	4.46	3.05	4.46	5.54	5.54	n/a	Left	Yes	No
S3	IVH Diversion	29	65	pIVH / Shunt	5.27	n/a	n/a	5.27	8.49	n/a	n/a	n/a	No	No
S4	IVH Diversion	27	27	pIVH / Shunt	5.67	5.67	5.67	5.67	4.35	4.35	4.35	Left	Yes	Yes
S5	IVH Diversion	22	38	Grade 4 / Shunt	4.95	6.01	6.66	6.66	6.99	6.99	6.80	Left	Yes	Yes
S6	IVH	28–29	32	pIVH / VAD	8.00	8.00	5.80	8.00	8.34	8.34	7.10	Neutral	Yes	Yes

	Diversion													
S7	IVH Diversion	26	30	pIVH / VAD	5.34	5.34	4.14	5.34	5.57	5.57	5.07	Neutral	Yes	Yes
S8	IVH Diversion	24	34	Grade 4 / EVD+Shnt	4.76	n/a	4.76	4.76	5.22	n/a	5.22	Neutral	No	No
S9	IVH Diversion	28	48	Grade 4 / Shunt	4.26	4.26	n/a	4.26	3.36	3.36	n/a	Left	No	No
S10	IVH Diversion	24	44	Grade 4 / Shunt	4.73	n/a	4.73	4.73	4.57	n/a	4.57	Left	No	No
S11	IVH Diversion	23	29	pIVH / Shunt	7.50	7.50	5.09	7.50	8.86	8.86	7.14	Neutral	Yes	Yes
S12	IVH Diversion	31	35	Grade 4 / Shunt	6.16	6.63	6.16	6.63	5.77	5.77	5.20	Neutral	Yes	Yes
S13	IVH Diversion	24	38	Grade 3 / Shunt	4.86	n/a	4.86	4.86	8.37	n/a	8.37	Left	No	No
S14	IVH Diversion	28	32	pIVH / Shunt	2.71	n/a	2.71	2.71	5.55	5.55	n/a	Left	No	No
S15	IVH Diversion	37	37	pIVH / Shunt	7.89	7.31	7.89	7.89	6.29	6.05	6.29	Neutral	Yes	Yes
S16	IVH Diversion	25	41	Grade 4 / Shunt	5.88	n/a	n/a	5.88	6.39	n/a	n/a	n/a	No	No
S17	IVH Diversion	24	33	Grade 3 / VAD	4.62	5.14	4.62	5.14	7.04	4.99	7.04	Right	Yes	Yes
S18	IVH Diversion	35	44	IVH / VP Shunt	6.66	5.64	6.66	6.66	7.97	5.50	7.97	Neutral	Yes	Yes
S19	IVH Diversion	24	27	IVH / VP Shunt	4.56	3.87	4.56	4.56	4.09	3.32	4.09	Neutral	Yes	Yes

Summary of bilateral availability: Axial bilateral: Controls 14/14 (100%), IVH no surgery 6/9 (67%), IVH diversion 12/19 (63%). Sagittal bilateral: Controls 13/14 (93%), IVH no surgery 6/9 (67%), IVH diversion 11/19 (58%). Sensitivity analysis (larger available axial side): Controls mean 3.59 mm, IVH no surgery 4.67 mm, IVH diversion 5.61 mm; $F[2,39] = 14.14$, $p < 0.0001$.

Index (mm) = subject-level ChP thickness used in primary analysis (Supplementary Table 3). Larger side = max(L, R) used in sensitivity analysis; where only one side measurable, that value is used. Blue rows = Control; Yellow rows = IVH without surgery; Red rows = IVH with CSF diversion.
